# Surveillance of HIV Transmitted Drug Resistance in Latin America and the Caribbean: A Systematic Review and Meta-Analysis

**DOI:** 10.1371/journal.pone.0158560

**Published:** 2016-06-29

**Authors:** Santiago Avila-Rios, Omar Sued, Soo-Yon Rhee, Robert W. Shafer, Gustavo Reyes-Teran, Giovanni Ravasi

**Affiliations:** 1 Centre for Research in Infectious Diseases, National Institute of Respiratory Diseases, Mexico City, Mexico; 2 Clinical Research Section, Huésped Foundation, Buenos Aires, Argentina; 3 Department of Medicine, Stanford University, Stanford, California, United States of America; 4 Pan American Health Organization (PAHO), Washington DC, United States of America; University of Maryland School of Medicine, UNITED STATES

## Abstract

**Background:**

HIV transmitted drug resistance (TDR) remains at moderate level in Latin America and the Caribbean (LAC). However, different epidemiologic scenarios could influence national and sub-regional TDR levels and trends.

**Methods and Findings:**

We performed a systematic review of currently available publications on TDR in antiretroviral treatment-naïve adults in LAC. Ninety-eight studies published between January 2000 and June 2015 were included according to critical appraisal criteria and classified by sub-region: Brazil (50), Mesoamerica (17), Southern Cone (16), Andean (8) and Caribbean (7). From these, 81 studies encompassing 11,441 individuals with data on DR mutation frequency were included in a meta-analysis. Overall TDR prevalence in LAC was 7.7% (95% CI: 7.2%-8.2%). An increasing trend was observed for overall TDR when comparing 2000–2005 (6.0%) and 2006–2015 (8.2%) (p<0.0001), which was associated with significant NNRTI TDR increase (p<0.0001). NRTI TDR decreased (4.5% vs. 2.3%, p<0.0001). NNRTI TDR increase was associated mainly with K101E, K103N and G190A. NRTI TDR decrease was associated mainly with M184V, K70R and T215Y. All sub-regions reached moderate overall TDR levels. The rapid increase in TDR to all antiretroviral classes in the Caribbean is notable, as well as the significant increase in NNRTI TDR reaching moderate levels in the Southern Cone. NRTI TDR was dominant in 2000–2005, mainly in the Caribbean, Mesoamerica and Brazil. This dominance was lost in 2006–2015 in all sub-regions, with the Southern Cone and the Caribbean switching to NNRTI dominance. PI TDR remained mostly constant with a significant increase only observed in the Caribbean.

**Conclusions:**

Given the high conceptual and methodological heterogeneity of HIV TDR studies, implementation of surveys with standardized methodology and national representativeness is warranted to generate reliable to inform public health policies. The observed increasing trend in NNRTI TDR supports the need to strengthen TDR surveillance and programme monitoring and evaluation in LAC.

## Introduction

LAC has some of the oldest national universal access antiretroviral treatment (ART) programs among low- and middle-income countries (Brazil, Argentina, Mexico, Chile, Costa Rica to name a few), and many of these programs introduced access to mono- and dual therapies in the 90’s. By the end of 2014, the estimated ART coverage in people living with HIV reached 46%, the highest worldwide among low- / middle-income countries (44% in the Caribbean and 47% in Latin America) [1]. In addition, the World Health Organization (WHO) recently published an early-release guideline recommending that ART should be initiated in everyone living with HIV at any CD4 cell count and number of people initiating ART in future years is therefore expected to increase [2].

In recent years, most LAC countries have been adopting the WHO/UNAIDS Treatment 2.0 approach as a strategy for enhancing and upgrading HIV care and treatment policies and programmes, including the optimization of ART regimens, simplification of diagnostic algorithms, expansion of point-of-care diagnostics, improvement in efficient procurement of HIV medicines and leveraging community engagement to increase HIV treatment uptake [3, 4]. Treatment 2.0 has supported the adoption of a public health approach to HIV care and treatment, aiming at improving the effectiveness and sustainability of the response towards the post-2015 Fast-Track targets, in particular the so-called “90-90-90” [4–6]. However, problems such as recurring stock-outs of antiretroviral (ARV) drugs, late detection of HIV infection, gaps in linkage and retention in care, and suboptimal retention in care and viral suppression on ART are observed in many LAC countries [7, 8].

In this context, the prevention and surveillance of HIV drug resistance (HIVDR) in persons initiating and on ART are critical to maintain current achievements and guarantee the effectiveness and sustainability of care and treatment and the progress towards the “90-90-90” targets. Since the launching of the “3 by 5” initiative in 2003, the WHO has been coordinating worldwide efforts to contain HIVDR and developed a public health strategy based on a comprehensive package of indicators and surveys to gauge the levels and patterns of resistance in different populations and generate strategic information for decision-making and quality improvement [9, 10].

Since 2007, the implementation of the WHO strategy in LAC mainly focused on EWI monitoring at ART sites, while HIVDR surveillance based on WHO surveys has been implemented in few countries and in some cases with substantial methodological adaptations, mainly due to limited feasibility of WHO protocols in the context of concentrated epidemics [11]. According to the WHO HIVDR Global Report 2012, the estimated prevalence of transmitted drug resistance (TDR) in recently infected individuals increased between 2003 and 2010 in low-/middle-income countries (mainly Africa) [10]. Nevertheless, evidence of HIVDR transmission in LAC is widely documented in the scientific literature, even though the vast majority of HIVDR surveillance studies did not apply the WHO recommended methodology and used the term TDR for drug resistance detected both in recently and chronically infected individuals. A systematic review on (TDR), which included 48 HIVDR surveys conducted between 1995 and 2009, showed TDR prevalence of 6.9% with no significant change over-time [12]. In a recent review including 1,922 publicly available *pol* sequences from LAC, an overall TDR prevalence of 7.7% was found for the period of 1996–2009 [13]. Another recent systematic review on TDR, including 26 studies performed in LAC between 1993 and 2008, showed a similar TDR prevalence of 6.3% [14]. Furthermore, the most recent comprehensive meta-analysis including 5,628 sequences from LAC (median sample year 2007) reported an overall TDR level of 7.6%, with increasing yearly change in odds of TDR due to NNRTI TDR (p<0.001) [15].

Even though TDR appears to remain at moderate levels (5–15% according to WHO threshold classification) [16] in most LAC countries, mutation patterns seem to vary regionally and over time, probably depending on changing national ART guidelines and prescribing practices. In addition, considering significant national and sub-regional differences in LAC regarding ART roll out, ART coverage, proportion of individuals under ART that reach undetectable viral load levels, ARV drug prescribing practices, national ARV guidelines and available ARV drugs, it is likely that HIVDR emergence and transmission presents sub-regional and national specificities. Given this scenario, the purpose of this work is to provide a systematic updated review of currently available published data on TDR in ART-naïve adults in LAC, including both chronically and recently infected subjects, and all target populations. Differently from previously published reviews, we included regional literature in Spanish and Portuguese, and highlighted sub-regional and national TDR characteristics. In addition, we included a comprehensive sub-regional meta-analysis of all studies with HIVDR mutation frequency data highlighting differences in the epidemiologic scenarios within the region.

## Methods

### Systematic literature review

We included data from population-based surveys assessing drug resistance in ARV naïve adults (hereby referred to as TDR, assessed in both chronically and recently infected ARV naïve individuals), as well as from other HIVDR or HIV diversity research studies and data analyses, performed in Latin America and the Caribbean and published in peer reviewed journals, or presented as abstracts at relevant international meetings (International AIDS Society and International AIDS Conference, CROI, and International HIVDR Workshop) between January 2000 and June 2015.

**Fig 1:**
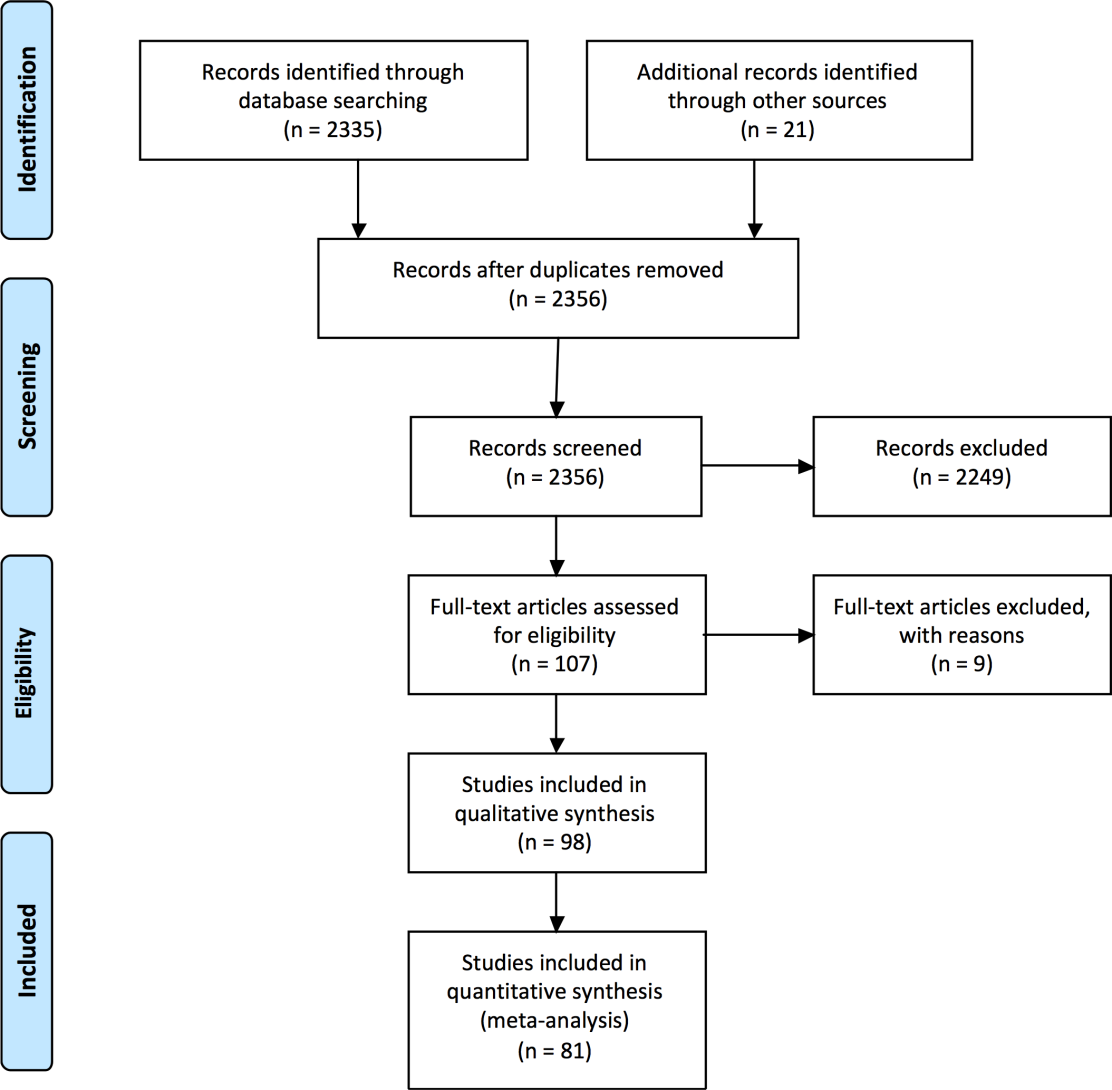
PRISMA flow diagram. The derivation of study sets meeting qualitative and quantitative inclusion criteria is shown.

Literature abstraction was performed using NCBI-NIH PubMed (Medline of the National Center for Biotechnology Information of the National Institute of Health). Literature abstraction of conference abstracts was performed from conference-specific search engines available on-line (International AIDS Society and International AIDS Conference, and CROI) or from conference reports published in peer reviewed journals (International HIVDR Workshop Abstract book, annually published on *Antiviral Therapy*). Search terms can be consulted at S1 Text. The PRISMA flow diagram [17] for study selection is shown in Fig 1 and the PRISMA checklist is available in S2 Table.

Articles and abstracts were included in the review only if the survey/study population was clearly described in terms of gender, age and type of population; the size of the cohort was stated; the type of “transmitted drug resistance” was defined (resistance in recently infected subjects vs. in subjects of unknown time of infection); the time of sampling was reported; and laboratory methods for genotyping were described (this criteria was not applied for conference abstracts). Only TDR in adults was considered for this review. Studies with less than 10 participants were excluded and studies that were re-published with expanded sample size were only included once. Except for children, all types of populations were considered, including blood donors, pregnant women, key/vulnerable populations and mixed populations. Key and vulnerable populations included men who have sex with men, sex workers, people who inject drugs, inmates and individuals in the army. We defined “mixed” populations as those including individuals from both general and key populations without differentiation. The definition of recent infection differed between studies according to one or more of the following criteria: serological testing algorithms for recent HIV seroconversion, based on BED enzyme immunoassay (EIA) and/or limited antigen avidity (LAg) EIA, plus viral load and/or CD4+ T cell count tests; record of seroconversion date with common EIA; negative or indeterminate Western Blot; record of acute retroviral syndrome; or WHO criteria (age <25 years, no pregnancies if women).

In our review we characterized and discussed drug resistance data according to the following sub-regional distribution: Andean Region (Bolivia, Colombia, Ecuador, Peru and Venezuela); Southern Cone (excluding Brazil; Argentina, Chile, Paraguay and Uruguay); Brazil (whole country and by sub-region); Mesoamerica (Costa Rica, El Salvador, Guatemala, Honduras, Mexico, Nicaragua, and Panama); and the Caribbean. In order to be able to compare reported TDR prevalence among studies, when available, mutations were revised and TDR prevalence recalculated according to the WHO list of mutations for TDR surveillance, except for conference abstracts, where no data on individual HIVDR mutations was usually available [18]. TDR levels to any drug class, as well as to individual drug classes (NRTI, NNRTI, PI), were classified according to the WHO recommended thresholds for transmitted resistance: low <5%; moderate 5–15%; high >15% [16]. Even though data were not usually collected according to WHO methods, these thresholds have been used traditionally to link TDR estimates with public health implications and possible actions.

### Meta-analysis

All publications in which data on individual drug resistance (DR) mutation frequency was available to recalculate TDR levels according to the WHO list of mutations for HIV TDR surveillance [18] were included in a meta-analysis comparing TDR per sub-region, and before and after 2005 (for a complete table of studies see S1 Appendix). For studies for which HIV sequences were publicly available, TDR was re-calculated using the Calibrated Population Resistance (CPR) tool [19], available online at the Stanford HIV Database [20, 21]. When sequences were available, additional studies on HIV diversity and subtype distribution in ART-naïve individuals that were not included in the general review were considered for the meta-analyses, using the CPR tool to estimate TDR. When sequences were not available, but HIVDR mutation frequency was, TDR prevalence was re-calculated considering only mutations included in the WHO list. Analyses of simultaneous TDR mutations to different ARV families were not performed. Surveys were grouped according to the mid-point year of the sampling period: 2000–2005 vs. 2006–2015. The year 2005 was arbitrarily chosen to equally divide available TDR results, but it can also be considered a time point dividing surveys applied before and after the general implementation of broad access programmes to ART in the region. Regional trends in LAC were also explored dividing the sampling period in three: 2000–2005 vs. 2006–2010 vs. 2011–2015. Trends on NRTI vs. NNRTI TDR dominance were analysed defining dominance as a 2.5% or higher TDR prevalence. Fisher’s exact tests or Chi-squared tests were used to compare TDR levels and mutation frequencies between groups using GraphPad Prism v6.0b. HIV subtype sub-regional distribution was also calculated, including all publications with available data and according to subtyping reported in each study. For studies with publicly available sequences, HIV subtype was re-assessed using REGA HIV-1 Subtyping tool (v3) [22]. Each study included in the systematic review and meta-analysis was individually revised by local ethics committees. All data included in the present study was used exclusively for HIV drug resistance surveillance.

## Results

A total of 98 studies: 79 articles and 19 abstracts, published between January 2000 and June 2015, were included according to critical appraisal criteria: 50 (51.0%) from Brazil, 17 (17.3%) from Mesoamerica, 16 (16.3%) from the Southern Cone, 8 (8.2%) from the Andean region and 7 (7.1%) from the Caribbean (Table 1). Selected articles/abstracts reported results from a total population of 18,320 individuals: 8,740 (47.7%) from Mesoamerica, 6,167 (33.7%) from Brazil, 1,848 (10.1%) from the Southern Cone, 714 (3.9%) from the Caribbean and 851 (4.6%) from the Andean Region (Table 1). Six surveys reported TDR results for recently infected individuals, 80 for chronically infected individuals of unknown time of infection and 12 for both, adding up to a total of 110 TDR results (Table 1). Most reports on TDR in recently infected individuals were performed in Brazil (8/19, 42%), followed by Mesoamerica and the Southern Cone (5/19, 26% each).

**Table 1:** Characteristics of participants in the surveys included in the present review per region.

	Caribbean	Mesoamerica	Andean Region	Brazil	Southern Cone	All
**Individuals included [n (%)]**	714 (3.9)	8740 (47.7)	851 (4.6)	6,167 (33.7)	1,848 (10.1)	**18,320 (100)**
**Sample size [median (range)]**	95 (27–152)	161 (24–1655)	63 (20–326)	74 (12–444)	77 (16–284)	**85 (12–1655)**
**Type of publication [n (%)]**						
Published articles	7 (100.0)	12 (70.6)	7 (87.5)	42 (84.0)	11 (68.8)	**79 (80.6)**
Conference abstracts	0 (0.0)	5 (29.4)	1 (12.5)	8 (16.0)	5 (31.3)	**19 (19.4)**
**Median sample year [n (%)]**						
2000–2005	2 (28.6)	7 (41.2)	3 (37.5)	21 (42.0)	7 (43.8)	**40 (40.8)**
2006–2015	5 (71.4)	10 (58.8)	5 (62.5)	29 (58.0)	9 (56.2)	**58 (59.2)**
**Recency of Infection [n (%)]**						
RI	0 (0.0)	2 (11.8)	0 (0.0)	1 (2.0)	3 (18.8)	**6 (6.1)**
Chronically infected	7 (100.0)	12 (70.6)	7 (87.5)	42 (84.0)	11 (68.8)	**79 (80.6)**
Both	0 (0.0)	3 (17.6)	1 (12.5)	7 (14.0)	2 (12.5)	**13 (13.3)**
**Type of population [n (%)]**						
Mixed	7 (100.0)	16 (94.1)	6 (75.0)	36 (72.0)	13 (81.3)	**78 (79.6)**
Pregnant women	0 (0.0)	0 (0.0)	0 (0.0)	6 (12.0)	1 (6.3)	**7 (7.1)**
Blood donors	0 (0.0)	0 (0.0)	0 (0.0)	3 (6.0)	0 (0.0)	**3 (3.1)**
Key and other vulnerable populations ^a^	0 (0.0)	1 (5.9)	2 (25.0)	5 (10.0)	2 (12.5)	**10 (10.2)**
**Total of publications [n (%)]**	**7 (7.1)**	**17 (17.3)**	**8 (8.2)**	**50 (51.0)**	**16 (16.3)**	**98 (100)**

^a^ This includes men who have sex with men, female sex workers, people who inject drugs, inmates and individuals in the army.

As far as type of population, the majority of surveys (78 surveys; 79.6% of total) were performed in mixed population recruited at either HIV reference or ART sites, voluntary counseling and testing (VCT) sites, or from available stored samples at local or national reference laboratories. Seven surveys were performed in pregnant women, and 3 in blood donors. Ten surveys were performed in key populations or other vulnerable groups: 6 in men who have sex with men (MSM); 2 in female sex workers (FSWs, one combined with MSM); 1 in injecting drug users (IDUs); 1 in inmates; and 1 in army soldiers (Table 1).

The overall median sample size was 85 (range 12–1,655). Median sample size was larger for the Mesoamerican (161) and the Caribbean (95) regions. Twenty-six studies had less than 47 participants, the previously WHO recommended size per year for threshold TDR analyses. The median sampling time-period was 2 years (range 1 to 7). For 41% of studies the median sampling year was between 2000 and 2005. For 59% of studies, the median sampling year was between 2006 and 2015.

### Regional considerations

For the meta-analysis, a total of 81 studies (from the original 98 reviewed) were included encompassing 11,441 individuals: 9 from the Caribbean, 10 from Mesoamerica, 7 from the Andean Region, 44 from Brazil and 11 from the Southern Cone (Table 2). Derived from this analysis, the overall TDR prevalence in LAC for the complete period of analysis (2000–2015) was 7.7% (95% CI: 7.2%-8.2%). An increasing temporal trend was observed for overall TDR in LAC when comparing the 2000–2005 (6.0%) and the 2006–2015 (8.2%, p<0.0001, Table 2) period. This increase in TDR was associated with a significant increase in NNRTI TDR (p<0.0001), which was also observed in most of the sub-regions (Table 2). The prevalence of NRTI TDR, however, did not increase between the 2000–2005 and 2006–2015 periods (4.5% vs. 3.8%, p = 0.11). In fact, it decreased between 2000–2005 and 2011–2015 (4.5% vs. 2.3%, p<0.0001) (Table 2 and S1 Table). Overall, for the 2000–2005 period, NRTI dominance (defined as a 2.5% or higher TDR prevalence) was observed when comparing NRTI vs. NNRTI TDR (4.5% vs. 1.8%). This NRTI dominance was lost in the 2006–2015 period as NNRTI TDR increased and NRTI TDR decreased (Table 2). NRTI dominance for 2000–2005 was also evident for the Caribbean, Mesoamerica and Brazil, and was lost in 2006–2015. NNRTI dominance was observed only in the Southern Cone during the 2006–2015 period (Table 2).

**Table 2:** PDR meta-analysis for the LAC region, 2000–2015.

	Complete cohort		2000–2005		2006–2015		p value ^a^
	%	(95% CI)	%	(95% CI)	%	(95% CI)	
Caribbean	n = 1,004		n = 415		n = 589		
Any ARV Drug	8.5	(6.8, 10.4)	3.6	(2.0, 5.9)	11.9	(9.3, 14.8)	<0.0001
NRTI	5.4	(4.1, 7.0)	3.4	(1.9, 5.6)	6.8	(4.9, 9.1)	0.0223
NNRTI	4.9	(3.6, 6.4)	0.2	(0.0, 1.3)	8.1	(6.1, 10.7)	<0.0001
PI	0.8	(0.3, 1.6)	0.0	(0.0, 0.9)	1.4	(0.6, 2.7)	0.0240
Mesoamerica	n = 3,663		n = 560		n = 3,103		
Any ARV Drug	7.3	(6.5, 8.2)	9.3	(7.0, 12.0)	7.0	(6.1, 7.9)	NS
NRTI	3.8	(3.2, 4.5)	7.9	(5.8, 10.4)	3.1	(2.5, 3.8)	<0.0001
NNRTI	3.5	(3.0, 4.2)	5.0	(3.3, 7.1)	3.3	(2.7, 4.0)	NS
PI	1.4	(1.1, 1.9)	1.6	(0.7, 3.0)	1.4	(1.0, 1.9)	NS
Andean	n = 677		n = 410		n = 267		
Any ARV Drug	5.7	(4.1, 7.7)	3.9	(2.2, 6.3)	8.2	(5.2, 12.2)	0.0251
NRTI	3.0	(1.8, 4.6)	2.4	(1.2, 4.4)	3.7	(1.8, 6.8)	NS
NNRTI	2.1	(1.2, 3.5)	1.0	(0.3, 2.5)	3.7	(1.8, 6.8)	0.0233
PI	2.2	(1.3, 3.7)	2.0	(0.8, 3.8)	2.6	(1.1, 5.3)	NS
Brazil	n = 4,954		n = 1,548		n = 3,406		
Any ARV Drug	8.4	(7.7, 9.2)	6.7	(5.5, 8.1)	9.1	(8.2, 10.2)	0.0044
NRTI	4.4	(3.8, 5.0)	4.8	(3.8, 6.0)	4.2	(3.2, 4.9)	NS
NNRTI	3.5	(3.0, 4.0)	1.2	(0.7, 1.9)	4.5	(3.8, 5.2)	<0.0001
PI	2.1	(1.7, 2.6)	1.8	(1.2, 2.6)	2.3	(1.8, 2.9)	NS
Southern Cone	n = 1,143		n = 567		n = 576		
Any ARV Drug	6.2	(4.9, 7.8)	4.2	(2.7, 6.2)	8.1	(6.1, 10.7)	0.0068
NRTI	2.7	(1.9, 3.8)	2.5	(1.4, 4.1)	3.0	(1.7, 4.7)	NS
NNRTI	3.7	(2.7, 4.9)	1.8	(0.8, 3.2)	5.6	(3.8, 7.8)	0.0008
PI	1.4	(0.8, 2.3)	0.7	(0.2, 1.8)	2.1	(1.1, 3.6)	NS
Complete Region	n = 11,441		n = 3,500		n = 7,941		
Any ARV Drug	7.7	(7.2, 8.2)	6.0	(5.3, 6.9)	8.2	(7.7, 8.9)	<0.0001
NRTI	4.0	(3.7, 4.4)	4.5	(3.8, 5.2)	3.8	(3.4, 4.2)	NS
NNRTI	3.6	(3.2, 3.9)	1.8	(1.4, 2.3)	4.2	(3.8, 4.7)	<0.0001
PI	1.7	(1.5, 2.0)	1.4	(1.0, 1.8)	1.8	(1.5, 2.1)	NS

^a^ Fisher’s exact test comparing the 2000–2005 vs. 2006–2015 periods. PDR, pre-antiretroviral treatment drug resistance; ARV, antiretroviral; NRTI, nucleoside reverse transcriptase inhibitors; NNRTI, non-nucleoside reverse transcriptase inhibitors; PI, protease inhibitors; CI, confidence interval; NS, non significant (p>0.05).

**Fig 2:**
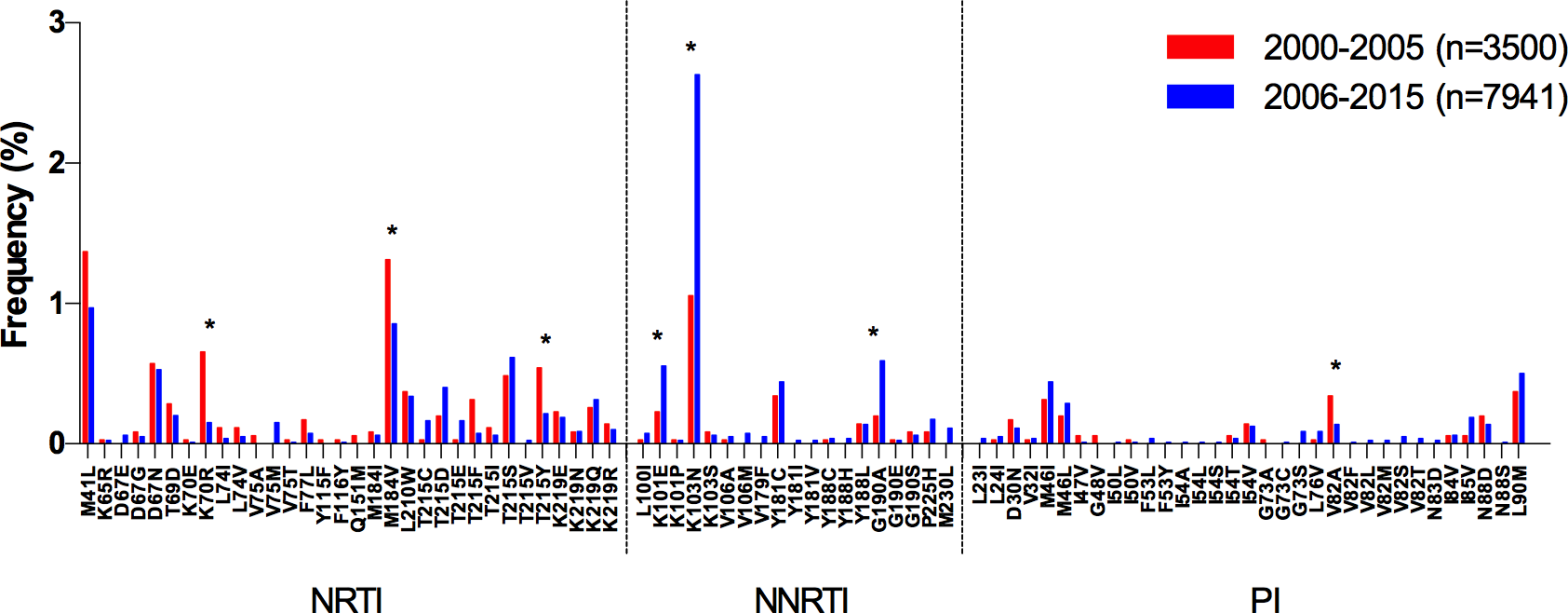
Drug resistance mutation frequency comparison, 2000–2005 vs. 2006–2015, for the whole LAC region. Drug resistance mutation frequency is shown for the studies included in the meta-analysis. Only surveillance mutations included in the WHO list are shown, ordered by drug family. *Significant differences (p<0.05), Fisher’s exact test. NRTI–nucleoside reverse transcriptase inhibitors; NNRTI–non-nucleoside reverse transcriptase inhibitors; PI–protease inhibitors.

The overall increase in NNRTI TDR was associated with a significant increase in the frequency of K101E (p = 0.03), K103N (p<0.0001), and G190A (p = 0.008; Fig 2). The overall decrease in NRTI TDR was associated with a significant decrease in the frequency of M184V (p = 0.03) and the thymidine analog mutations (TAMs) K70R (p<0.0001) and T215Y (p = 0.007) (Fig 2). PI TDR remained mostly stable along the study period, with a significant increase only observed in the Caribbean (p = 0.02). The following sections describe sub-regional temporal trends in the prevalence of NRTI and NNRTI-associated TDR and in the prevalence of specific drug-resistance mutations.

**Fig 3:**
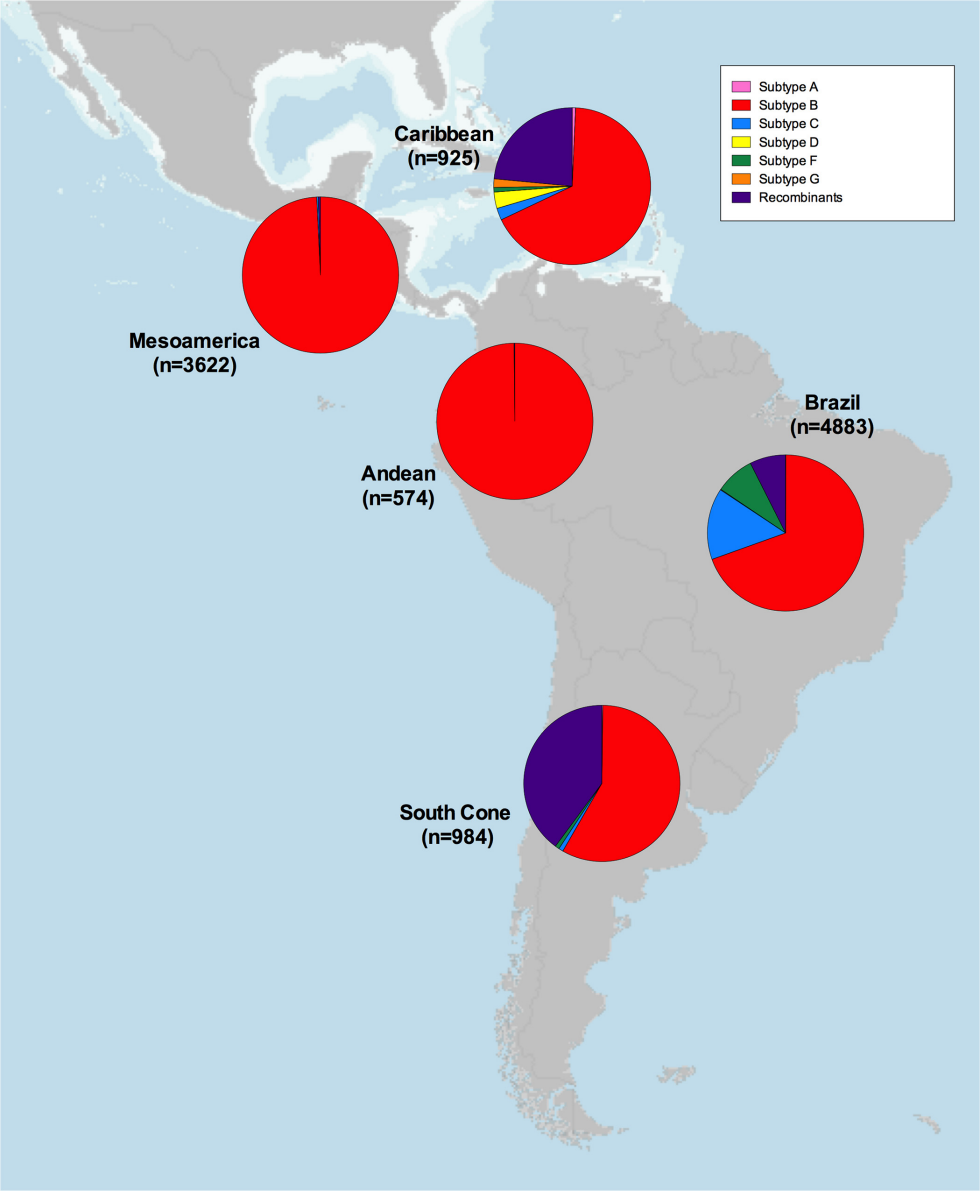
HIV subtype distribution in the LAC region. HIV subtype distributions are shown for the studies included in the meta-analysis by sub-region. Map obtained from [23] (public domain).

HIV subtype distribution was also estimated as part of the meta-analysis, excluding 7 studies not including data on HIV subtype prevalence. As expected, HIV subtype B was highly prevalent in the region, especially in the Mesoamerican (99.2%) and Andean (99.8%) areas. However, non-B subtypes were frequent in the Caribbean (32.7%), Brazil (30.5%) and the Southern Cone (41.9%; Fig 3). Subtype C (14.8%) and F (8.1%) were prevalent in Brazil, while recombinants were frequently observed in the Southern Cone (39.9%) and the Caribbean (23.5%).

### Caribbean

Information on HIV TDR in the Caribbean is still very limited. Published data include 7 surveys: 4 from Cuba, 1 from the Dominican Republic, and 2 from Jamaica, all of them in mixed population (Table 3).

**Table 3:** Publications on HIV PDR in the Caribbean, 2000–2015.

Authors	Publication Year	Abstract / Paper	Country and Area	Sampling Period	Median Sampling Year	Study Population	N	PDR Prevalence (%)			
								Any Drug Class	NRTI	NNRTI	PI
Ruibal-Brunet et al.	2001	Paper (M)	Cuba	1999	1999	Mixed	27	7.4	7.4	0	0
Pérez et al.	2007	Paper (M)	Cuba (Havana)	2003	2003	Mixed	250	3.6	3.6	0	0
Pérez et al.	2013	Paper (M)	Cuba	2007–2011	2009	Mixed	152	12.5	8.6	6.6	2.6
Machado et al.	2013	Paper (M)	Cuba	2009–2011	2010	Mixed	183	**15.5**	11.5	10.9	1.5
Myers et al.	2011	Paper (M)	Dominican Republic (Santo Domingo)	2007–2010	2009	Mixed	103	7.8	1.0	6.8	0
Hamilton et al.	2012	Paper (M)	Jamaica	2009	2009	Mixed	72	9.7	5.6	5.6	1.4
Barrow et al.	2013	Paper (M)	Jamaica	2011	2011	Mixed	79	10.1	1.3	8.9	0

PDR, pre-antiretroviral treatment drug resistance; NRTI, nucleoside reverse transcriptase inhibitors; NNRTI, non-nucleoside reverse transcriptase inhibitors; PI, protease inhibitors; RI, recently infected; M, included in meta-analysis.

In Cuba, moderate TDR prevalence to NRTIs (7.4%) was reported in a small survey performed in 1999 in 27 ARV-naïve subjects as part of a multi-country UNAIDS HIVDR study [24]. Nevertheless, a second survey performed in Havana in 2003 in 250 ARV naïve individuals showed low prevalence of NRTI TDR, and no evidence of TDR to NNRTIs and PIs [25]. This study was later expanded to 401 participants, including samples from 2007–2011 observing a significant increase in TDR in recently infected individuals compared to 2003 (14.8 vs. 3.8%; OR 3.9, 95% CI 1.5–17.0, p = 0.02). This increase was mostly due to NRTI TDR [26]. Additionally, a survey including 200 patients enrolled between 2009 and 2011 reported an overall TDR prevalence of 21.5%, with K103N and M184V being the most frequent TDR mutations [27]. In all, these results suggest a significant increase in TDR in Cuba, mainly due to NRTI TDR, reaching high or borderline high levels in the most recent surveys.

Data from a more recent HIVDR survey performed in Santo Domingo, Dominican Republic, in 2007–2010 in 103 ART-naïve adults enrolled in a prospective observational cohort study, showed moderate TDR to any ARV class (7.8%), 6.8% TDR to NNRTIs and 1.0% to NRTI [28].

A recent survey in Kingston, Jamaica, including 79 amplifiable samples reported a moderate overall TDR level (10.1%) [29]. This work contrasts with an older study reporting high (29%) overall TDR levels in Jamaica [30] and a recent small study reporting high level TDR in pregnant women attending antenatal clinics in Kingston [31].

**Fig 4:**
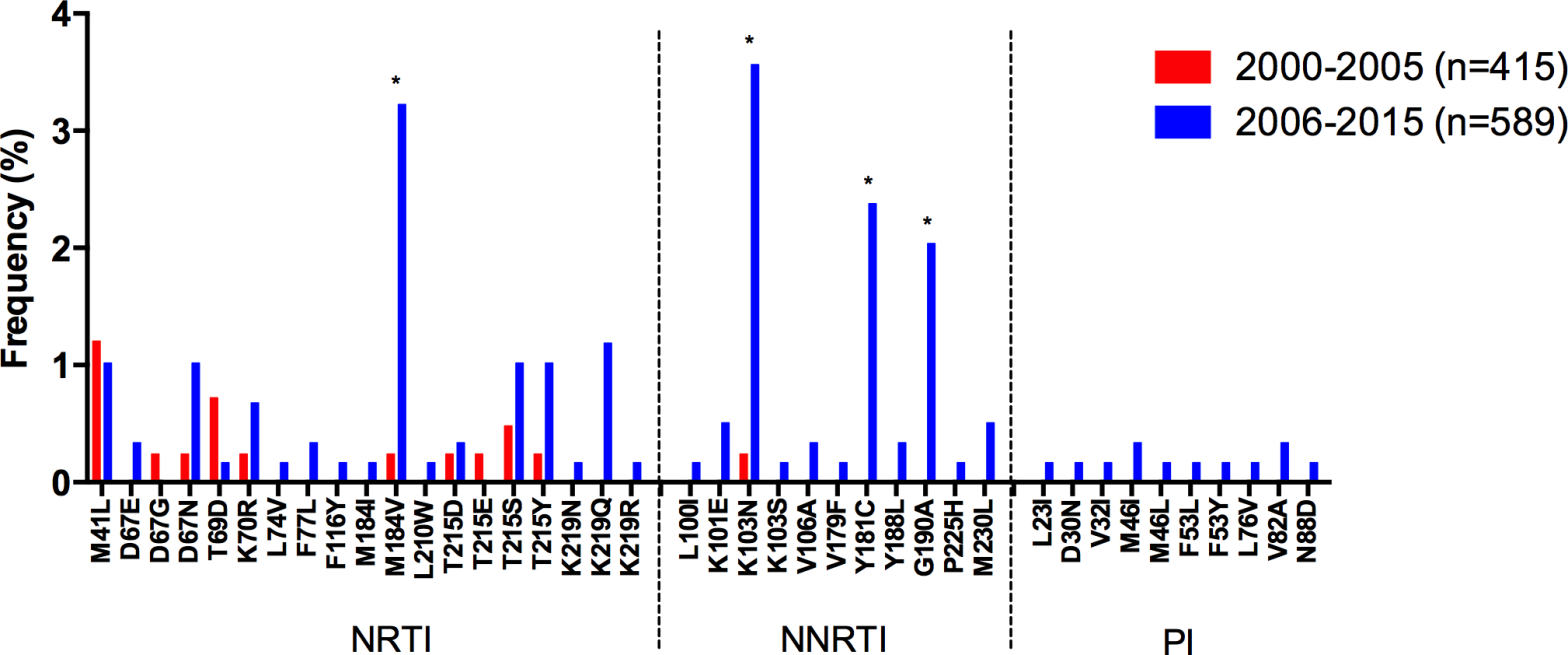
Drug resistance mutation frequency comparison, 2000–2005 vs. 2006–2015, for the Caribbean. Drug resistance mutation frequency is shown for the studies included in the meta-analysis. Only surveillance mutations included in the WHO list are shown, ordered by drug family. *Significant differences (p<0.05), Fisher’s exact test. NRTI–nucleoside reverse transcriptase inhibitors; NNRTI–non-nucleoside reverse transcriptase inhibitors; PI–protease inhibitors.

After performing a meta-analysis from 9 studies [2 additional studies focused on HIV diversity in ART-naïve individuals with available sequences were included: [32, 33]], encompassing 1,004 individuals, the global TDR observed in the Caribbean for the whole study period was 8.5% (95% CI: 6.8–10.4; Table 2). The most prevalent DR mutations included M41L (1.1%), M184V (2.0%) for NRTI, and K103N (2.2%), Y181C (1.4%) and G190A (1.2%) for NNRTI (Fig 4).

Of note, an important and significant increase in TDR was observed in the region, when comparing 2000–2005 with 2006–2015 (p<0.0001; Table 2) or 2000–2005 with 2011–2015 (p = 0.02; S1 Table), with a significant increase in TDR to all drug classes (Table 2). This increase in TDR was also associated with significant increase in the prevalence of several DR mutations, mainly M184V for NRTI; and K103N, Y181C and G190A for NNRTI. Remarkably, PI TDR was not observed during 2000–2005, appearing later in the 2006–2015 period, when PI DR mutations were observed at low frequencies in the population (Table 2, Fig 4).

### Mesoamerican Region

Information on TDR in the Mesoamerican region including Mexico and Central America was available from 17 surveys (2 multinational, 7 in Mexico, 1 in Guatemala, 2 in El Salvador, 3 in Honduras, 2 in Panama), most of them performed in mixed population, including 1 survey in MSM and FSW in Honduras (Table 4). No published data were available on HIV TDR in Costa Rica (data on Belize and Nicaragua were available only as part of a multi-national study).

**Table 4:** Publications on HIV PDR in the Mesoamerican Region, 2000–2015.

Authors	Publication Year	Abstract / Paper	Country and Area	Sampling Period	Median Sampling Year	Study Population	N	PDR Prevalence (%)			
								Any Drug Class	NRTI	NNRTI	PI
Murillo et al.	2011	Abstract	Honduras, Panama, El Salvador	2004–2007	2006	Mixed	388	8.5	4.6	5.4	0.5
Avila-Rios et al.	2015	Abstract	Mexico	2010–2014	2012	Mixed	1476	8.5	3.4	3.0	2.9
			Guatemala				1180	7.1	1.8	4.6	1.1
			Panama				238	12.2	3.8	9.2	0.8
			Nicaragua				222	12.7	6.4	5.5	1.7
			Honduras				294	9.9	2.0	6.5	2.0
			Belize				100	**19**	1.0	**18.0**	1.0
Ahumada-Ruiz et al.	2009	Paper	Panama (Panama City and other districts)	2004–2005	2005	Mixed	53	0	0	0	0
Castillo et al.	2011	Paper	Panama (Panama City)	2008–2010	2009	Mixed, RI (WHO criteria)	47	12.7	8.5	4.2	2.1
Lloyd et al.	2008	Paper (M)	Honduras (San Pedro Sula)	2002–2003	2003	Mixed	215	6.5	5.6	4.2	1.4
Murillo et al.	2010	Paper (M)	Honduras (Tegucigalpa)	2002–2003	2003	Mixed	121	14.0	11.6	12.4	5.0
			Honduras (Tegucigalpa, San Pedro Sula and other areas of the country)	2004–2007	2006	Mixed	176	5.1	1.7	3.4	0.6
						Mixed, RI (BED EIA)	24	**20.8**	12.5	**16.7**	0
Avila-Rios et al.	2015	Abstract	Honduras (Tegucigalpa, San Pedro Sula, Choluteca, La Ceiba)	2013–2014	2014	Mixed	294	9.9	2.0	6.5	2.0
						Mixed, RI (BED + LAg Avidity EIA)	52	13.5	1.9	7.7	3.8
Avila-Rios et al.	2015	Paper (M)	Guatemala (Guatemala City and most departments)	2010–2013	2012	Mixed	1084	7.3	1.8	4.9	1.0
						Mixed, RI (BED + LAg Avidity EIA)	72	9.7	0	9.7	0
Murillo et al.	2012	Paper (M)	El Salvador	2008	2008	FSW and MSM	145	9.4	4.2	5.9	0.8
Holguín et al.	2013	Paper (M)	El Salvador	2011	2011	Mixed	88	5.7	2.3	2.3	1.4
Valle-Bahena et al.	2006	Paper (M)	Mexico (North-East)	2001–2003	2002	Mixed	36	2.8	2.8	0	0
Escoto-Delgadillo et al.	2005	Paper (M)	Mexico (Western region)	2002–2003	2003	Mixed	96	12.5	9.4	5.2	2.1
Viani et al.	2007	Paper (M)	Mexico (Tijuana, Baja California)	2003–2005	2004	Mixed	41	2.5	2.5	0	0
Bertagnolio et al.	2012	Paper (M)	Mexico (Central Mexico)	2004	2004	Mixed, RI (WHO criteria)	47	6.4	6.4	0	0
Rodriguez Diaz et al.	2007	Abstract	Mexico (Central Mexico)	2004–2005	2005	Mixed	403	7.4	6.2	2.2	1.2
Silva et al.	2007	Abstract	Mexico (Mexico City and 2 other states)	2004–2008	2006	Mixed	193	4.7	1.6	2.6	1.6
Avila-Rios et al.	2011	Paper (M)	Mexico (Several states)	2005–2010	2008	Mixed	1655	6.8	4.2	1.9	1.8

MSM, men who have sex with men; FSW, female sex workers; EIA, enzyme immunoassay; LAg, limiting antigen; PDR, pre-antiretroviral treatment drug resistance; NRTI, nucleoside reverse transcriptase inhibitors; NNRTI, non-nucleoside reverse transcriptase inhibitors; PI, protease inhibitors; RI, recently infected; M, included in meta-analysis.

In Honduras, a large study performed in 2002–2003, including individuals from Tegucigalpa and San Pedro Sula, reported 6.5% and 14.0% overall TDR respectively, suggesting important differences in HIV management within the country [34]. Most TDR cases were observed both for NRTI and NNRTI. A later study performed in 2004–2007 in 176 individuals from mainly Tegucigalpa and San Pedro Sula reported a moderate TDR level to any ARV drug (5.1%), with higher NNRTI TDR (3.4%) compared to NRTI (1.7%) and PI (0.6%) [35]. This study also reported high-level TDR in recently infected individuals defined using the BED incidence test (n = 24, 20.8%), associated with NRTI and NNRTI. The most recent survey including 294 individuals enrolled in 5 HIV centers in Tegucigalpa, San Pedro Sula, La Ceiba and Choluteca in 2013–2014 reported moderate overall TDR levels (9.9%), mostly associated with NNRTI (6.5%), compared to NRTI (2.0%) and PI (2.0%) [36]. These two later surveys suggest an increasing trend in time in overall TDR in Honduras, mainly due to an increase in NNRTI resistance, which is consistent with the wide use of this ARV drug family in first-line ART regimens.

In El Salvador, a study on female sex workers (n = 47) and MSM (n = 98) in 2008, reported moderate TDR levels of 10.3% and 9.0%, respectively [37]. Overall, TDR to any ARV drug was 9.4% with NNRTI TDR (4.9%) being higher than NRTI (1.8%) and PI (1.0%). Interestingly, TDR in recently infected individuals defined with the BED incidence test (n = 19, 10.5%) remained similar to that observed in chronically infected individuals. Another recent study in mixed population carried out from dried blood spots from 88 ART-naïve individuals in 2011 showed lower levels of TDR (5.7% to any ARV drug), remaining however at the moderate range [38]. In this study, NRTI and NNRTI TDR prevalence were similar (2.3%) and higher than PI TDR (1.4%).

In Panama, an earlier survey carried out in 2004–2005 in 53 ART-naïve individuals from Panama City (44%) and other districts found no evidence of TDR [39]. Nevertheless, a more recent WHO TDR threshold survey in 47 recently infected ART-naïve individuals from Panama City, performed in 2008–2010 reported a moderate level of TDR to any ARV drug (12.7%), with higher NRTI TDR (8.5%) compared to NNRTI (4.2%) and PI (2.1%) [40].

A multinational study performed in mixed population from 2004 to 2007, including individuals from Honduras (n = 200), El Salvador (n = 119) and Panama (n = 69) reported TDR levels of 7.0%, 9.2% and 11.5% respectively (overall 8.5%), confirming moderate TDR levels in the region [41].

In Guatemala, a study performed in 2010–2011 in 145 ART-naïve from one reference center receiving patients from all over the country found an overall TDR level of 8.3%, mainly associated with NNRTI TDR (6.9%), which was significantly higher than NRTI (0.7%) and PI (0.7%) TDR [42]. This study was later expanded to 1,085 participants from 2010 to 2013, finding similar results, with 7.3% TDR prevalence to any ARV drug, and higher NNRTI TDR (4.9%) compared to NRTI (1.8%) and PI (1.0%) TDR [43]. This study also estimated moderate level TDR in recently infected individuals identified using a multi-assay algorithm including two incidence tests, viral loads and CD4+ T cell counts (n = 72, 9.7%). These TDR cases in recently infected individuals included only NNRTI TDR cases, suggesting a temporal increase in NNRTI TDR in the country consistent with the predominant use of efavirenz-containing first line regimens.

A recent multinational survey performed from 2010 to 2014, including chronically infected, ART-naïve individuals from most Mesoamerican countries reported moderate TDR levels in Mexico (n = 1476, 7.7%), Guatemala (n = 1180, 7.1%), Panama (n = 238, 12.2%), Nicaragua (n = 222, 14.9%), Honduras (n = 294, 9.9%) and high TDR level in Belize (n = 100, 19.0%) [44]. NNRTI TDR was higher compared with NRTI and PI in Guatemala (4.6% vs. 1.8% and 1.1%), Panama (9.2% vs. 3.8% and 0.8%), Honduras (6.5% vs. 2.0% and 2.2%) and Belize (18.0% vs. 1.0% and 1.0%), and NRTI TDR was significantly higher in Nicaragua compared to Panama, Belize, Honduras, Mexico and Guatemala.

Information on TDR in Mexico was available from 7 surveys, performed in different geographic areas of the country, and all of them in mixed population (Table 4). Two older contemporary studies performed between 2001 and 2003 reported different overall TDR levels in two regions of the country: 12.5% (n = 96) in the Western region, including the city of Guadalajara [45] and 2.8% (n = 36) in the Northeast including the city of Monterrey [46]. In both cases, most TDR cases were associated with NRTI. A study performed from 2003 to 2005 in the city of Tijuana, in the Northwest region at the border with the USA, reported low TDR levels (n = 41, 2.5%), associated with NRTI [47]. More recent studies have reported moderate TDR levels in the country. A study performed in 2004–2005 in 403 chronically infected individuals from 9 cities in Central Mexico showed moderate TDR levels (7.4%) to any ARV drug, with higher NRTI TDR levels (6.2%) compared to NNRTI (2.2%) and PI (1.2%) [48]. Similarly, a large national study including 1655 chronically infected individuals from 12 Mexican states, performed from 2005 to 2010 also reported moderate TDR levels (6.8%) to any ARV drug, with higher NRTI TDR (4.2%) compared to NNRTI (1.9%) and PI (1.8) TDR [49]. This study also reported an increasing temporal trend in NNRTI and PI TDR. Moreover, a WHO threshold survey performed in three voluntary counseling testing sites in Mexico City (n = 47) in 2004 reported moderate levels of NRTI TDR and low-level NNRTI and PI TDR [50], consistent with other recent studies showing higher NRTI TDR in the country [48, 49]. These observations contrast with a survey performed in 2004–2008 in 193 chronically infected, ART-naïve individuals from 3 centers in Mexico City and 2 in other states, which found low overall TDR level (4.7%), with higher NNRTI TDR (2.6%) compared to NRTI (1.6%) and PI (1.6%) TDR [51].

**Fig 5:**
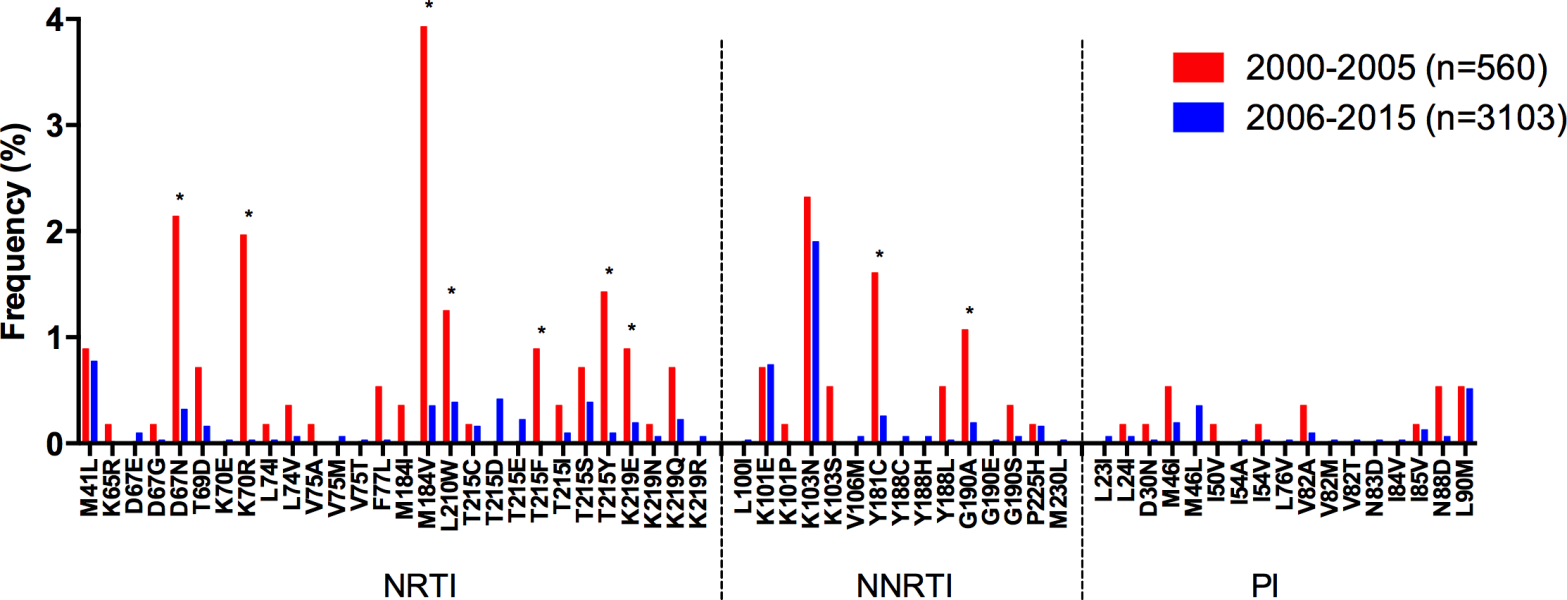
Drug resistance mutation frequency comparison, 2000–2005 vs. 2006–2015, for the Mesoamerican region. Drug resistance mutation frequency is shown for the studies included in the meta-analysis. Only surveillance mutations included in the WHO list are shown, ordered by drug family. *Significant differences (p<0.05), Fisher’s exact test. NRTI–nucleoside reverse transcriptase inhibitors; NNRTI–non-nucleoside reverse transcriptase inhibitors; PI–protease inhibitors.

The meta-analysis in the Mesoamerican region including 10 studies and 3,663 individuals showed an overall TDR level of 7.3 (95% CI: 6.5–8.2), with comparable NRTI (3.8%) and NNRTI (3.5%) TDR levels, and low PI TDR (1.4%, Table 2). No significant trends in time in overall TDR in the region were observed. However, contrasting with the other regions, a significant decrease in NRTI TDR was observed when comparing the 2000–20005 and 2006–2015 periods (p<0.0001, Table 2). This decreasing trend was associated with reductions in frequency of several DR mutations including D67N, K70R, M184V, L210W, T215F, T215Y, and K219E (p<0.05; Fig 5). Although a significant decrease in frequency of Y181C and G190A was observed (p<0.01), no significant trends were observed in NNRTI TDR. Contrasting to other regions, no changes were observed in the frequency of K103N. For this region, the most prevalent TDR mutations for the whole study period were K103N (2.0%), M184V (0.9%), M41L (0.8%), and D67N (0.6%).

### Andean Region

Information on TDR in the Andean Region was available from 6 surveys performed in mixed population (4 from Venezuela, 1 from Colombia and 1 from Peru) and 2 in MSM (1 from Peru and 1 from Peru and Ecuador; Table 5). No published data was available for Bolivia.

**Table 5:** Publications on HIV PDR in the Andean Region, 2000–2015.

Authors	Publication Year	Abstract / Paper	Country and Area	Sampling Period	Median Sampling year	Study Population	N	PDR prevalence (%)			
								Any drug class	NRTI	NNRTI	PI
Diaz Granados et al.	2010	Paper (M)	Colombia (Bogotá / Cundinamarca, Valle del Cauca, Antioquia, Atlantico / Bolivar, Santander, Caldas / Risaralda)	2006–2008	2007	Mixed	103	5.8	2.9	4.9	1.0
Lama et al.	2006	Paper (M)	Peru (Lima, Sullana, Piura, Arequipa, Iuitos, Pucallpa)	2002–2003	2003	MSM	326	3.4	2.1	0.6	1.8
						RI MSM (Detuned EIA)	33	3.0	3.0	3.0	3.0
Guanira et al.	2009	Abstract	Peru (Lima, Arequipa, Ica, Sullana) and Ecuador (Guayaquil)	2006	2006	MSM	117	4.3	0.6	2.6	0.6
Soria et al.	2011	Paper (M)	Peru (Lima)	2007–2009	2008	Mixed	96	1.0	0	1.0	0
Delgado et al.	2001	Paper (M)	Venezuela	1999	1999	Mixed	31	3.2	3.2	0	0
Bouchard et al.	2007	Paper (M)	Venezuela (Caracas, Central Venezuela)	2003	2003	Mixed	20	5.0	5.0	0	0
Rangel et al.	2009	Paper (M)	Venezuela (Caracas)	2004–2007	2006	Mixed	63	11.0	9.5	3.2	1.6
Castillo et al.	2009	Paper (M)	Venezuela (Caracas)	2008	2008	Mixed	62	6.5	3.2	1.6	1.6

MSM, men who have sex with men; EIA, enzyme immunoassay; PDR, pre-antiretroviral treatment drug resistance; NRTI, nucleoside reverse transcriptase inhibitors; NNRTI, non-nucleoside reverse transcriptase inhibitors; PI, protease inhibitors; RI, recently infected; M, included in meta-analysis.

In Venezuela, older studies performed mainly in Caracas, reported low-level or borderline moderate overall TDR: 3.2% (n = 31, 1999) [52]; 5.0% (n = 20, 2003) [53], all NRTI TDR cases. More recent studies showed an increase to moderate TDR levels: 11.0% (n = 63, 2004–2007) [54]; 6.5% (n = 62, 2008) [40]. TDR cases were mostly associated with NRTI; however, NNRTI and PI TDR were also observed (Table 5).

In Peru, the only study performed in mixed population in Lima from 2007 to 2009, showed low TDR prevalence to any ARV class (1%). Nevertheless, this study was performed in a cohort of ART-naïve individuals eligible for ART (<200 CD4+ cells/μl), most likely to have been infected prior to the launching of the national ART programme or at its initial scale up, and with low chance of exposure to drug resistant HIV strains due to the limited ART coverage at the population level [55]. Two surveys in MSM mainly from Lima, but also including individuals from Sullana, Arequipa, Iquitos, Piura, Pucalipa and Ica, performed at two different time periods, showed low overall TDR levels with no evidence of increasing trends in time: 3.4% (n = 359, 2002–2003) [56]; 4.3% (n = 117, 2006) [57]. Both studies reported individual TDR to the tree drug families at low levels.

The only study in Colombia, performed from 2006 to 2008 in 103 individuals from six regions of the country (Bogotá/Cundinamarca, Valle del Cauca, Antioquía, Atlántico/Bolívar, Santander and Caldas/Risaralda), reported moderate TDR levels (5.8%): 2.9% to NRTI, 4.9% to NNRTI and 1.0% to PI [58].

**Fig 6:**
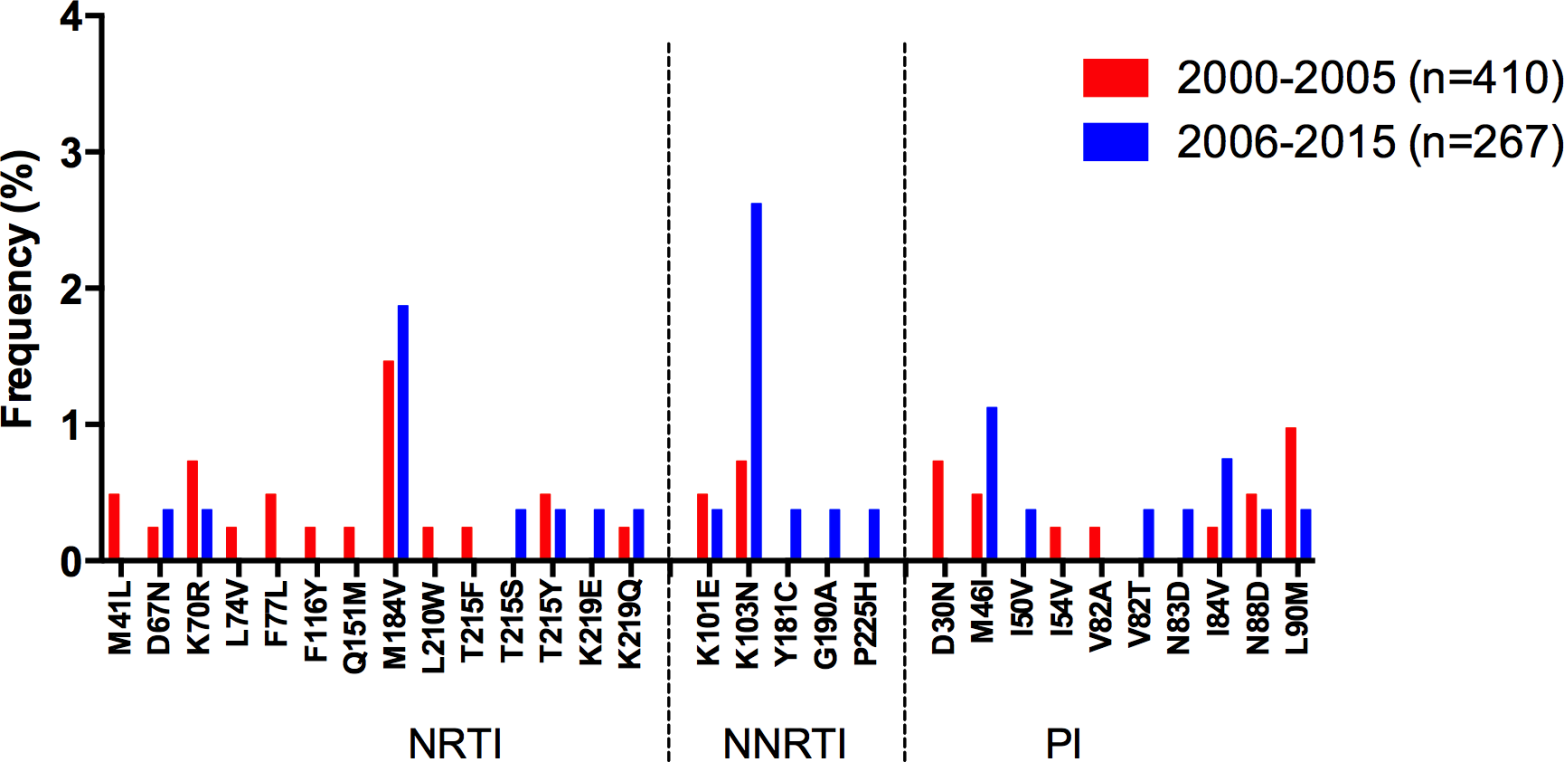
Drug resistance mutation frequency comparison, 2000–2005 vs. 2006–2015, for the Andean region. Drug resistance mutation frequency is shown for the studies included in the meta-analysis. Only surveillance mutations included in the WHO list are shown, ordered by drug family. *Significant differences (p<0.05), Fisher’s exact test. NRTI–nucleoside reverse transcriptase inhibitors; NNRTI–non-nucleoside reverse transcriptase inhibitors; PI–protease inhibitors.

The meta-analysis of 7 studies including 667 individuals from the Andean Region showed a global TDR level of 5.7 (95% CI: 4.1–7.7), with comparable levels of NRTI (3.0%), NNRTI (2.1%) and PI (2.2%) TDR (Table 2). A significant increase in overall TDR (p = 0.0251) associated with NNRTI resistance was observed in the region. This observation may be explained by a trend of increasing frequency of K103N (p = 0.0558) and the appearance of viruses with the Y181C, G190A, and P225H mutations in the 2006–2015 period at low frequency (Fig 6). For this region, M184V (1.6%) and K103N (1.5%) were the most prevalent TDR mutations.

### Brazil

Information on HIV TDR in Brazil was available from 50 surveys and, considering the size of the country and the regional epidemiological and diversity in health sector response to HIV and access to health care, TDR data are presented from national surveys (7 studies) and separately according to the 5 macro-regions of the country: Southeast (23 surveys), South (8 surveys), Central-West (7 surveys), Northeast (5 surveys). No surveys have been performed in the Northern region (Table 6).

**Table 6:** Publications on HIV PDR in Brazil, 2000–2015.

Authors	Publication Year	Abstract / Paper	Macro-region	Sampling Period	Median Sampling Year	Study Population	N	PDR Prevalence (%)			
								Any Drug Class	NRTI	NNRTI	PI
Brindeiro et al.	2003	Paper (M)	Brazil (multiple sites)	2001	2001	Mixed	356	4.9	2.4	0.3	2.3
Sa-Ferreira et al.	2007	Paper (M)	Brazil (multiple sites)	2000–2004	2002	Blood donors	74	1.4	1.4	0	0
Sprinz et al.	2009	Paper (M)	Brazil (multiple sites)	2007	2007	Mixed	387	5.2	1.8	3.6	1.0
Inocencio et al.	2009	Paper (M)	Brazil (multiple sites)	2007–2008	2008	Mixed	210	6.7	1.9	2.9	2.4
Bermudez-Aza et al.	2011	Paper (M)	Brazil (multiple sites)	2008–2009	2009	MSM	99	**21.4**	**15**	5.5	3.9
Alencar et al.	2013	Paper (M)	Brazil (multiple sites)	2007–2011	2009	Mixed	287	12.0	4.2	7.0	3.0
de Moraes Soares et al.	2014	Paper (M)	Brazil (multiple sites)	2009–2010	2010	Mixed	329	11.8	6.9	4.9	3.9
Dumans et al.	2002	Paper (M)	Southeast (Rio de Janeiro)	1998	1998	Blood donors	47	8.5	8.5	0	0
Maia Teixeira et al.	2006	Paper (M)	Southeast (Rio de Janeiro City)	1994–1997	1996	IDU	27	**22.0**	**22.0**	0	0
				1999–2001	2000	IDU	38	10.5	7.9	0	7.9
Varella et al.	2007	Paper (M)	Southeast (Rio de Janeiro City)	1999–2001	2000	Mixed	51	0	0	0	0
Varella et al.	2009	Paper (M)	Southeast (Rio de Janeiro City)	1999–2001	2000	Mixed	71	1.4	1.4	0	0
Pires et al.	2004	Paper (M)	Southeast (Rio de Janeiro City)	2000–2002	2001	Army	50	10.0	10.0	0	0
Eyer-Silva et al.	2005	Paper (M)	Southeast (Miracema, Rio de Janeiro State)	2001–2005	2003	Mixed	27	0	0	0	0
						Mixed, RI (<1 year seroconversion)	20	5.0	5.0	0	0
Tupinambás et al.	2013	Paper (M)	Southeast (Belo Horizonte; Rio de Janeiro State)	1996–2012	2004	MSM	64	14.1	7.8	6.2	4.7
						RI MSM (<1 year seroconversion)	35	**20.0**	14.3	8.6	5.7
Eyer-Silva et al.	2008	Paper (M)	Southeast (Saquarema, Santo Antonio de Pádua; Rio de Janeiro State)	2004–2006	2005	Mixed	50	2.0	2.0	0	0
Velasco-de-Castro et al.	2014	Paper ^a^	Southeast (Rio de Janeiro)	2005–2007	2006	Mixed	102	**15.7**	6.9	8.8	6.9
						Mixed, RI (BED EIA)	144	14.6	10.4	6.3	3.5
Pilotto et al.	2013	Paper (M)	Southeast (Rio de Janeiro)	2005–2008	2007	Pregnant women	197	10.7	5.6	2.0	3.0
Melo et al.	2011	Abstract	Southeast (Belo Horizonte, Ribeirao Preto; Rio de Janeiro State)	2009–2010	2010	Pregnant women	24	0	0	0	0
Teixeira et al.	2015	Paper (M)	Southeast (Rio de Janeiro)	2010–2012	2011	Pregnant women	174	8.7	1.2	8.1	0.6
Rodrigues et al.	2002	Abstract	Southeast (São Paulo City)	1995–2001	1998	Mixed	29	6.9	3.4	3.4	0
Barreto et al.	2006	Paper (M)	Southeastl (São Paulo City)	1998–2002	2000	Blood donors	280	4.3	2.9	1.1	0.7
						RI blood donors (STARHS)	55	10.9	7.3	0	5.5
Sucupira et al.	2007	Paper (M)	Southeast (Santos; São Paulo State)	1999–2000	2000	Mixed	65	**15.4**	13.8	1.5	3.1
						Mixed, RI (STARHS)	25	**32.0**	**20.0**	0	12.0
Gagliani et al.	2008	Abstract	Southeast (Santos; São Paulo State)	2000–2001	2001	Mixed	41	**41.5**	**21.9**	**29.2**	4.9
Gonsalez et al.	2007	Paper (M)	Southeast (São Paulo City)	2000–2006	2003	Mixed	123	5.7	3.3	2.4	2.4
Alcalde al.	2011	Abstract	Southeast (São Paulo City)	2002–2006	2004	Mixed	212	**25.0**	12.0	10.5	5.3
Sucupira et al.	2009	Abstract	Southeast (São Paulo City)	2002–2007	2005	Mixed, RI (STARHS)	174	**15.0**	4.0	11.0	4.0
Ambar et al.	2006	Abstract	Southeast (Santos; São Paulo State)	2006–2007	2007	Mixed	32	18.8	6.3	12.5	0
de Sa-Filho et al.	2009	Paper (M)	Southeast (Santos; São Paulo State)	2006–2008	2007	Mixed	30	**23.3**	10.0	13.3	3.3
Ferreira et al.	2013	Paper (M)	Southeast (São Paulo City, Campinas)	2008–2009	2009	Mixed	221	7.6	2.3	5.4	1.4
De Souza-Guimarães et al.	2015	Paper (M)	Southeast (São Paulo City)	2012–2014	2013	Mixed	186	11.2	3.8	5.9	2.2
Rodrigues et al.	2005	Paper (M)	South (Porto Alegre; Rio Grande do Sul State)	2004	2004	Mixed	87	3.1	1.0	2.1	0
Rodrigues et al.	2008	Abstract	South	2003–2006	2005	Mixed	444	8.6	3.2	5.4	0
Ferreira et al.	2008	Paper (M)	South (Curitiba; Paraná State)	2005–2006	2006	Mixed	57	7.0	1.8	5.2	0
Santos et al.	2011	Paper (M)	South (Rio Grande; Rio Grande do Sul State)	2005–2008	2007	Mixed	85	9.2	4.7	4.7	3.3
de Medeiros et al.	2011	Paper (M)	South (Porto Alegre; Rio Grande do Sul State)	2006–2007	2007	Mixed	99	8.1	3	3	2
Gräf et al.	2011	Paper (M)	South (Florianopolis; Santa Catarina State)	2008–2009	2009	Mixed	82	9.7	4.9	4.9	2.4
Gaspareto et al.	2012	Paper (M)	South (Maringá; Paraná State)	2009	2009	Mixed	48	4.2	2.1	0	2.1
Prellwitz et al.	2013	Paper (M)	South (Porto Alegre; Rio Grande do Sul State)	2009	2009	Inmates	31	6.5	0	3.2	3.2
Cardoso et al.	2010	Paper (M)	Central-West (Goiania; Goiás State)	2003	2003	Pregnant women	35	0	0	0	0
Cardoso et al.	2009	Paper (M)	Central-West (Goiania; Goiás State)	2007–2008	2008	Mixed	97	9.3	6.2	4.1	2.1
Bacelar et al.	2010	Abstract	Central-West (Goiás State)	2008–2009	2009	Pregnant women	53	5.7	0	3.8	1.9
Carvalho et al.	2011	Paper (M)	Central-West (Palmas; Tocantins State)	2008–2009	2009	Mixed	52	11.5	3.8	5.8	1.9
Ferreira et al.	2011	Paper (M)	Central-West (Cuiabá; Mato Grosso State)	2008–2009	2009	Mixed	92	5.4	3.3	1.1	1.1
da Silveira et al.	2011	Paper (M)	Central-West (Campo Grande; Mato Grosso do Sul State)	2008–2010	2009	Mixed	49	6.1	6.1	0	0
da Costa et al.	2013	Paper (M)	Central-West	2010–2011	2011	Pregnant women	12	**16.7**	8.3	0	8.3
de Medeiros et al.	2006	Paper (M)	Northeast (Recife; Pernambuco State)	2002–2003	2003	Mixed	84	3.6	3.6	0	0
Cavalcanti et al.	2012	Paper (M)	Northeast (Recife; Pernambuco State)	2007–2009	2008	Mixed	130	4.6	1.5	3.8	0.8
						Mixed, RI (BED EIA)	25	8.0	8.0	8.0	0
Arruda et al.	2011	Paper (M)	Northeast (Fortaleza; Ceará State)	2008–2009	2009	Mixed	74	9.5	4.1	2.7	4.1
Soares-Moura et al.	2015	Paper (M)	Northeast (Piauí State)	2011–2012	2012	Mixed	89	10.1	2.2	4.5	3.4
Soares-Moura et al.	2015	Paper (M)	Northeast (Maranhao State)	2012	2012	Mixed	106	3.8	1.9	1.9	0

MSM, men who have sex with men; IDU, intravenous drug users; EIA, enzyme immunoassay; STARHS, serologic testing algorithm for recent HIV seroconversion; PDR, pre-antiretroviral treatment drug resistance; NRTI, nucleoside reverse transcriptase inhibitors; NNRTI, non-nucleoside reverse transcriptase inhibitors; PI, protease inhibitors; RI, recently infected; M, included in meta-analysis.

#### National surveys

Since 2001, 7 national surveys have been performed in multiple sites from different regions in the country (6 in mixed population; 2 in blood donors and 1 in MSM). Surveys performed before or around 2005 showed low TDR prevalence (<5%) to any ARV class: 4.9% in the first HIVDR BResNet survey in 2001 including 13 centers across the country (n = 356) [59], and 1.4% in blood donors from the North, North-East and South-East Regions in 2000–2004 (n = 74) [60]. More recent surveys performed in mixed adult populations with HIV infection of unknown duration showed higher TDR levels to any ARV class: 5.2% in 2007 [61]; 6.6% in 2007–2008 [62]; 12.2% in 2007–2011 [63] and 11.8% in 2009–2010 [64]. Within these studies, three large national surveys performed at different time intervals show clear TDR trends in time: Brindeiro et al. (2001, n = 356, including 13 centers across the country) reported 4.9% to any ARV class, 2.4% NRTI, 0.3% NNRTI, and 2.3% PI [59]; Inocencio et al. (2007–2008, n = 210, including Sao Paulo, Rio de Janeiro, Salvador, Porto Alegre, Brasilia and Belem) 6.6% to any ARV class, 1.9% NRTI, 2.9% NNRTI, and 2.4% PI [62]; de Moraes-Soares et al. (2009–2010, n = 329, including Manaus, Salvador, Brasilia, Rio de Janeiro, Santos, Porto Alegre, Itajaí) 11.8% to any ARV class, 6.9% NRTI, 4.9% NNRTI, and 3.9% PI [64]. Thus, increasing trends in TDR can be observed for the three drug classes, but are especially evident for NRTI passing from low to moderate level. The only national HIVDR survey in MSM, was performed using 44 samples collected in 9 capital cities from all 5 Brazilian regions in 2008–2009, as part of a national respondent-driven-sampling-based HIV surveillance study, and showed 21.4% prevalence of TDR mutations in recently diagnosed individuals (15.0% to NRTIs; 5.5% to NNRTIs; and 3.9% to PIs) [65]. This higher TDR prevalence in recently infected MSM is a concern and will have to be confirmed and closely monitored in future surveys.

#### Southeast region

TDR data for the Southeast Region, including Minas Gerais, Rio de Janeiro and Sao Paulo States, are available from 23 surveys (12 in Rio de Janeiro State: 5 in mixed population, 1 in blood donors, 1 in IDUs, 1 in army soldiers, 3 in pregnant women and 1 in MSM; and 11 in São Paulo State: 10 in mixed population; 1 in blood donors).

In Rio de Janeiro State, most TDR surveys performed in mixed population before 2005 showed <5% prevalence of TDR mutations to any ARV class within Rio de Janeiro city [66, 67] and other municipalities in the state: Miracema [68], Saquarema, Santo Antonio de Pádua [69]. In contrast, a few of the older studies performed around the year 2000 in Rio de Janeiro showed moderate TDR levels associated with mostly NRTI in blood donors [70], individuals in the army [71] and IV drug users [72]. More recent surveys performed after 2005 have shown moderate and even threshold high levels of TDR to any ARV class: 10.7% in pregnant women in Rio de Janeiro [73], 14.1% in MSM in Belo Horizonte/Rio de Janeiro [74] and 15.7% in people seeking HIV diagnosis in voluntary counseling and testing sites in Rio de Janeiro [75]. TDR to NRTI was generally higher, and in the most recent studies, increasing NNRTI and PI TDR levels can be observed compared to older studies. An exception is a small study in 24 pregnant women performed in 2009–2010 in Rio de Janeiro that showed no evidence of TDR [76]. Of note, the most recent survey in 231 pregnant women performed in 2010–2012 in Rio de Janeiro showed high-level TDR to any ARV drug [77].

In Sao Paulo state, surveys on mixed population, performed in the city of Santos showed generally higher prevalence of TDR to any ARV class: 12.3% [1999–2000 [78]], 41.5% [2000–2001, [79]], 18.8% [2006–2007, [80]], 18.2% [2006–2008 [81]], in comparison with surveys based in the city of Sao Paulo: 6.9% [1995–2001 [81]], 4.3% [1998–2002 [82]], 5.7% [2000–2006 [83]], 2.9% [2008–2009, [84]]. Of note, a TDR survey in recently infected individuals performed in 2002–2007 in in Sao Paulo reported high threshold levels (15%) of TDR to any ARV drug, mainly associated with NNRTI, suggesting an increasing trend in TDR [78]. This observation is in agreement with another survey in Sao Paulo reporting increasing NNRTI and NRTI TDR levels from 2002 to 2010 reaching 17.7% and 16.5% respectively [85]. Moreover, the most recent survey on 226 ART-naïve individuals performed in 2012–2014 in Sao Paulo, reported moderate TDR levels (9.2%), with 4.6% NNRTI, 3.6% NRTI and 1.8% PI TDR [86]. Most surveys in Santos reported moderate levels of TDR both to NRTI and NNRTI, while surveys in Sao Paulo reported low levels of TDR to NRTI and NNRTI. In general, PI TDR was low.

Overall, increasing trends in TDR both in Rio de Janeiro and Sao Paulo can be observed, reaching high levels (>15%) in many of the most recent surveys, and with NRTI and NNRTI being the most affected drug classes.

#### South region

TDR data for the South Region of Brazil are available from 8 surveys (4 in Rio Grande do Sul State; 2 in Paraná State; 1 in Santa Catarina State, and 1 in the whole sub-region), 7 in mixed populations and 1 in inmates.

An increasing overall TDR trend can be observed comparing TDR surveys in Rio Grande do Sul State from 2004 to 2008, mainly based in the capital city Porto Alegre, from low (3.1%) to moderate (8.1%) levels [87–89] [87, 89–91]. Moderate overall TDR levels have also been reported in other southern capitals: Curitiba (7.0%) [92] and Florianapolis (9.7%) [93], mainly associated to low or moderate NRTI and NNRTI TDR levels. Additionally, a recent TDR threshold WHO survey in Maringá, Parana reported low overall TDR levels [94]. Of note, a recent survey in inmates in the Porto Alegre area reported high levels of TDR associated to an alarming proportion of virologic failure and high secondary drug resistance [95].

#### Central-west region

Seven TDR surveys have been performed in the Central-West Region in Goias, Mato Grosso, Mato Grosso do Sul and Tocantins States (4 in mixed population, 3 in pregnant women).

With the exception of a first survey performed in 2003 in Goiania, Goias State, in 35 pregnant women which showed no evidence of TDR mutations [96], all subsequent surveys performed in capital cities of the region showed moderate prevalence (5–15%) of TDR mutations to any ARV drug, 9.3% [97] and 5.7% [98] in Goiania, in mixed adult population and pregnant women respectively; 5.4% in Cuiabá, Mato Grosso State [99]; 6.1% in Campo Grande, Mato Grosso do Sul State [100]; and 11.5% in Palmas, Tocantins State [101]; all with similar sampling periods (2007–2010). In this region, most surveys showed low prevalence (<5%) to individual ARV classes, with the exception of two surveys performed in 2007–2008 in Goiania and in 2008–2010 in Campo Grande, which showed 6.2 and 6.1% of NRTI TDR respectively [97, 100] and a survey performed in 2008–2009 in Tocantins showing 5.8% NNRTI TDR [101]. The latest survey performed in 18 pregnant women from the Central-West region, showed high overall TDR level (33.3%), which is a concern for HIV management and mother to child transmission prevention [102].

#### Northeast region

In the Northeast Region (Maranhao, Piauí, Pernambuco and Ceará States), 5 surveys have been performed in mixed population, showing contrasting results in the different states. Low-level TDR to any drug class was observed in Recife, Pernambuco State: 3.6% in 2002–2003 (n = 84) [103] and 4.6% in 2007–2009 (n = 108) [104]; as well as in Maranhao State: 3.8% (n = 106) [105]. In contrast, moderate TDR level was observed in Fortaleza, Ceará State: 9.5% in 2008–2009 (n = 74) [106] and in Piauí State: 11.2% (n = 89) [107]. In all cases, <5% TDR to each individual ARV class was observed. Both in Maranhao and Piauí, the majority of TDR cases were observed in MSM and in most surveys NRTI TDR was higher or equal to NNRTI TDR, which was in turn higher than PI TDR.

#### General considerations

In general, an increasing trend in overall TDR can be seen in most regions of Brazil, reaching moderate levels in most capital cities in the South, Central-West and Northeast regions, with some exceptions in the Pernambuco and Maranhao States (Northeast Region) still reporting low TDR levels. This contrasts with the high TDR levels reported in the most recent surveys on the Rio de Janeiro and Sao Paulo macro urban centers at the Southeast region.

**Fig 7:**
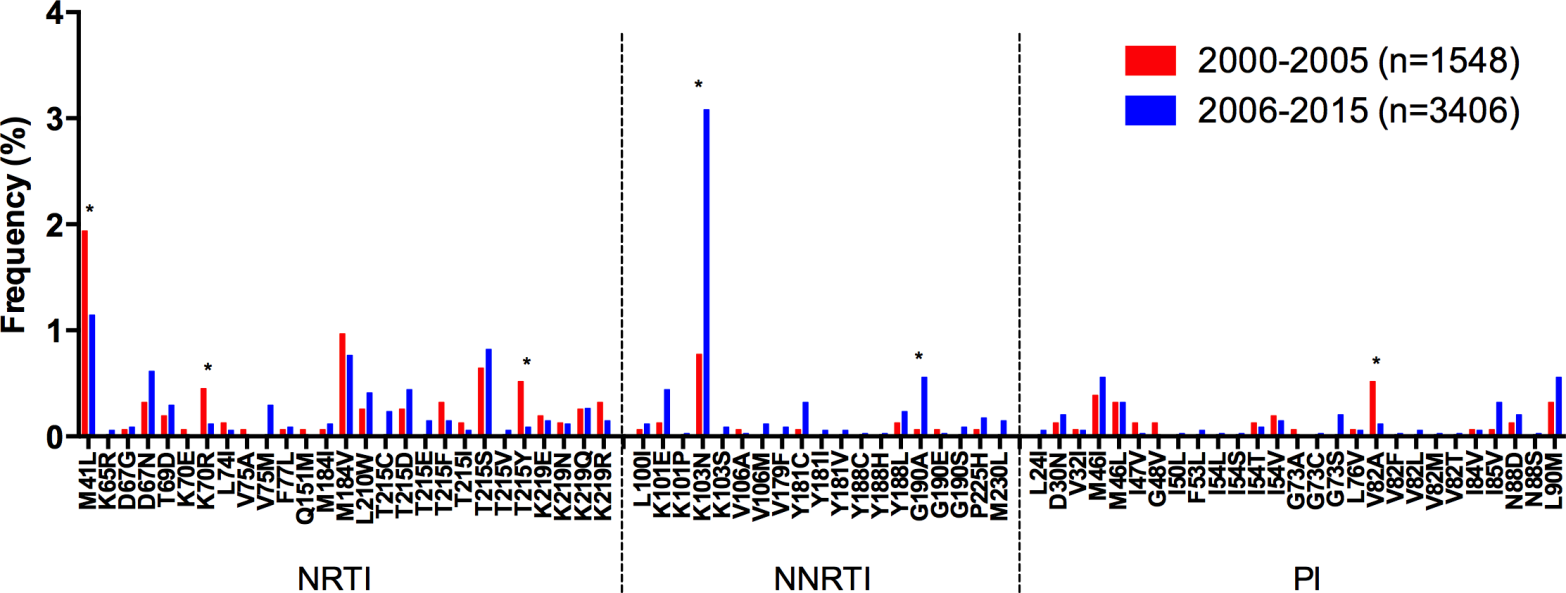
Drug resistance mutation frequency comparison, 2000–2005 vs. 2006–2015, for Brazil. Drug resistance mutation frequency is shown for the studies included in the meta-analysis. Only surveillance mutations included in the WHO list are shown, ordered by drug family. *Significant differences (p<0.05), Fisher’s exact test. NRTI–nucleoside reverse transcriptase inhibitors; NNRTI–non-nucleoside reverse transcriptase inhibitors; PI–protease inhibitors.

In the meta-analysis including 44 studies [three extra studies on HIV diversity in ART-naïve individuals with sequences available were included: [108–110]] and 4,954 individuals, a moderate overall TDR prevalence of 8.4% (95% CI: 7.7–9.2%) was observed for the country, with low level TDR to individual families: 4.4% for NRTI, 3.5% for NNRTI, and 2.1% for PI (Table 2). A significant temporal increase in overall TDR was observed (p = 0.0044), which was associated with a significant increase in NNRTI TDR (p<0.0001) and frequency of K103N (p<0.0001) and G190A mutations (p = 0.02, Fig 7). Although no significant change was observed in NRTI TDR prevalence, a significant decrease in the frequency of M41L (p = 0.04), T215Y (p = 0.008), and K70R (p = 0.046) mutations was observed (Fig 7). The most frequent TDR mutations for the whole country for the entire study period were M41L (1.4%), and K103N (2.4%). Considering the whole study period, overall TDR was moderate in all the macro-regions: 6.0% Northeast, 8.4% Central West, 7.4% South and 8.6% Southeast (data not shown). The Northeast showed a lower NRTI TDR prevalence than the Central West and the Southeast (p<0.05).

**Fig 8:**
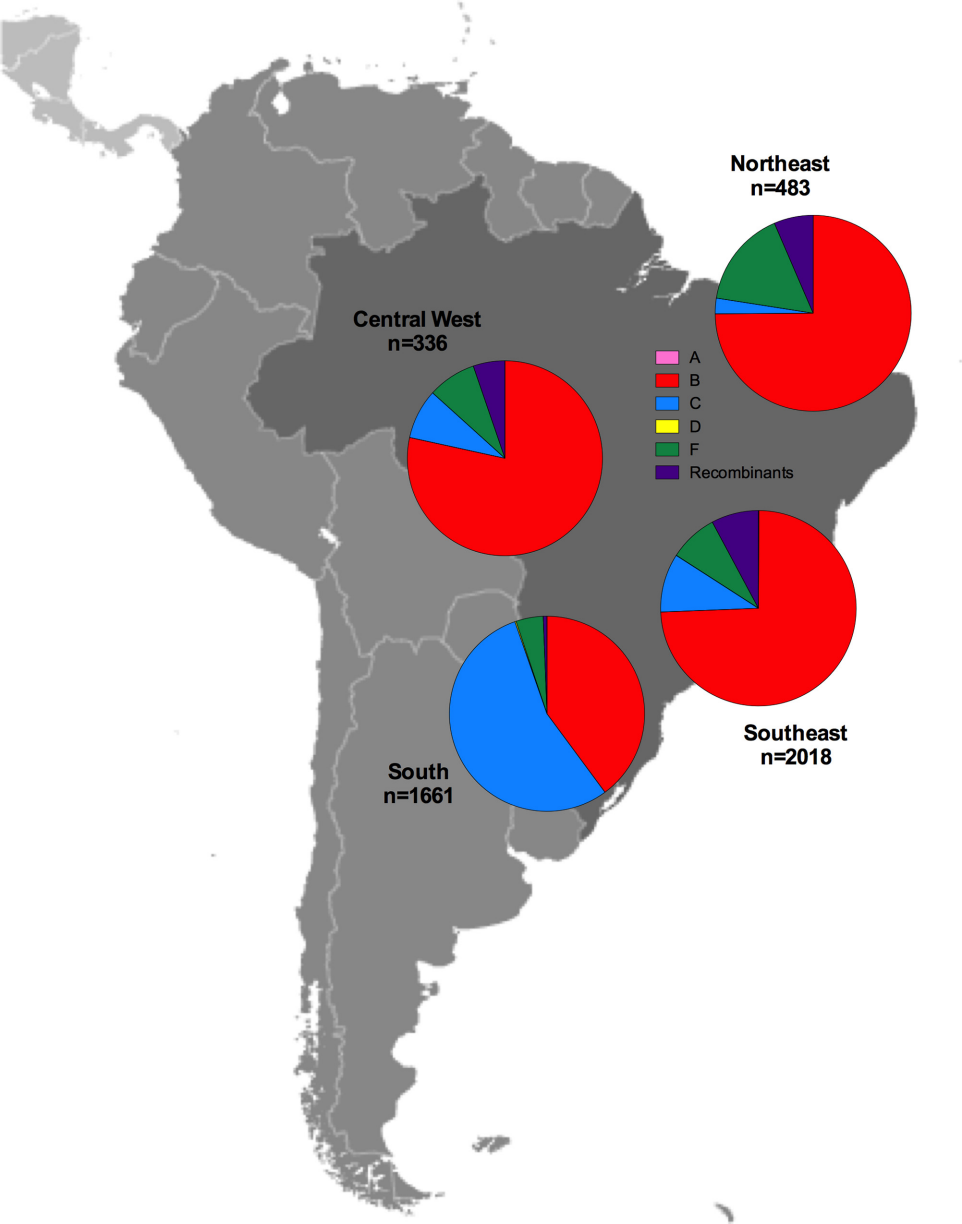
HIV subtype distribution in Brazil. HIV subtype distributions are shown for the studies included in the meta-analysis by macro-region. No studies were available for the North region. Map obtained from [111] (public domain).

Interestingly, subtype distribution in Brazil varied within the country when dividing by macro-region (Fig 8). Subtype B prevalence was higher in the Northeast (75.0%), Central West (78.4%) and Southeast (71.7%), but not in the South (39.9%), where subtype C predominated (54.8%, Fig 8). Also, subtype F had a higher prevalence in the Northeast (16.1%) and recombinants had a low frequency in the South compared to other regions.

### Southern Cone

Information on HIV TDR in the Southern Cone is available from 4 surveys performed in Chile (3 in mixed population and 1 in MSM) and 12 in Argentina (10 in mixed population, 1 in female sex workers and 1 in pregnant women). No published data were available on transmitted HIVDR in Paraguay and Uruguay (Table 7).

**Table 7:** Publications on HIV PDR in the Southern Cone, 2000–2015.

Authors	Publication Year	Abstract / Paper	Country and Area	Sampling Period	Median Sampling Year	Study Population	N	PDR Prevalence (%)			
								Any Drug Class	NRTI	NNRTI	PI
Kijak et al.	2001	Paper (M)	Argentina (Buenos Aires)	1997–2000	1999	Mixed	86	2.3	1.2	0	1.2
						Mixed, RI (previous negative ELISA)	13	15.4	7.7	0	7.7
Pando et al.	2007	Paper (M)	Argentina (Buenos Aires, Salta, Rosario, Córdoba, Mendoza, La Plata)	2000–2002	2001	FSW	16	12.5	12.5	6.3	0
Dilernia et al.	2007	Paper (M)	Argentina (Buenos Aires)	2003–2005	2004	Mixed	250	2.8	0.8	1.2	1.2
						Mixed, RI (Detuned EIA)	28	7.1	3.6	3.6	3.6
Petroni et al.	2006	Paper (M)	Argentina (Buenos Aires city, Rosario, Neuquen, Rio Negro)	2004–2005	2005	Mixed, RI (Negative or indeterminate WB or ARS or previous negative ELISA)	52	7.7	1.9	5.8	0
Patterson et al.	2007	Abstract	Argentina (Buenos Aires)	2005–2007	2006	Mixed	70	7.1	1.4	1.4	4.3
Parenti et al.	2008	Abstract	Argentina (Buenos Aires, Cordoba, Rosario)	2005–2007	2006	Mixed	85	2.4	1.2	1.2	0
Rubio et al.	2009	Abstract	Argentina (Buenos Aires)	2003–2005	2004	Mixed	284	3.2	1.4	0.4	1.4
				2006–2008	2007	Mixed	200	8	2.4	4.0	1.6
Pando et al.	2011	Paper (M)	Argentina (Buenos Aires, Santiago del Estero, Viedma, Rosario, Paraná, Córdoba, La Plata, Mendoza)	2006–2008	2007	Mixed	214	8.4	4.2	5.6	3.3
Zapiola et al.	2011	Abstract	Argentina (Buenos Aires)	2008	2008	Pregnant women	35	20	8.6	11.4	5.7
Arreseigor et al.	2010	Abstract	Argentina (Buenos Aires province)	2008–2009	2009	Mixed	47	6.4	4.2	0	2.1
Rodriguez-Rodriguez et al.	2013	Paper (M)	Argentina (Buenos Aires)	2010–2011	2011	Mixed	152	7.9	1.3	5.9	1.3
Cecchini et al.	2015	Paper (M)	Argentina (Buenos Aires)	2011–2013	2012	Mixed	91	12.1	4.4	7.7	2.2
Afani et al.	2005	Paper (M)	Chile (Santiago)	2001–2002	2002	Mixed	60	1.7	1.7	0	0
Ríos et al.	2007	Paper (M)	Chile (Central, North and South Region)	2000–2005	2003	Mixed	79	5	5	0	0
Acevedo et al.	2007	Paper (M)	Chile (Santiago)	2004–2005	2005	RI MSM (ARS or previous negative ELISA)	25	12	8	8	0
Afani et al.	2010	Paper (M)	Chile (Santiago)	2006–2008	2007	Mixed, RI (Previous negative ELISA or ARS)	74	2.7	1.4	1.4	0

MSM, men who have sex with men; FSW, female sex workers; EIA, enzyme immunoassay; WB, western blot, ARS, acute retroviral syndrome; PDR, pre-antiretroviral treatment drug resistance; NRTI, nucleoside reverse transcriptase inhibitors; NNRTI, non-nucleoside reverse transcriptase inhibitors; PI, protease inhibitors; RI, recently infected; M, included in meta-analysis.

In Chile, two similar studies before 2005 in mixed population with chronic infection showed ≤5% TDR prevalence: 1.7% (2001–2002, n = 60 individuals from Santiago) [112], 5.0% (2000–2005, n = 79 individuals mainly from the Central region [82%] including also individuals from the North [10%] and South [8%]) [113], all NRTI TDR cases. A more recent study including recently infected individuals from Santiago, during 2006–2008 (n = 74), also reported an overall low TDR prevalence of 2.7% [114]. Nevertheless, higher TDR prevalence was observed in a small survey performed in 2004–2005 in 25 recently infected MSM from Santiago, reporting 12% TDR to any ARV class, 8% to NRTI, 12% to NNRTI and 0% to PI [115]. These results suggest low level TDR prevalence in Chile with no evidence of increase in time.

In Argentina, surveys performed in Buenos Aires before 2005 generally reported low TDR prevalence in chronically infected individuals: 2.3% (n = 86, 1997–2000) [116]; 2.8% (n = 250, 2003–2005)[117]; 3.2% (n = 284, 2003–2005) [118]; while some of these older studies also reported moderate or borderline high TDR in recently infected individuals: 15.4% (n = 13, 1997–2000) [116]; 7.7% (n = 52, 2004–2005) [119]; 7.1% (n = 28, 2003–2005) [117], suggesting increasing TDR trends in time in Buenos Aires. Interestingly, a study including 16 female sex workers in 6 Argentinean cities, performed between 2000–2002, showed a high overall TDR level of 12.5% associated to NRTI and NNRTI [120]. Most of the more recent studies in mixed population in the Buenos Aires province have reported moderate TDR levels: 7.1% (n = 70, 2005–2007) [121]; 6.1% (n = 65, 2006–2007) [122]; 8.0% (n = 200, 2006–2008) [118]; 6.4% (n = 47, 2008–2009) [123]; 7.9% (n = 152, 2010–2011) [124]. Moreover, a larger, multi-site study including 214 individuals from Buenos Aires, Santiago del Estero, Viedma, Rosario, Paraná, Córdoba, La Plata and Mendoza cities, enrolled in 2006–2008, reported an overall TDR prevalence of 8.4% [125], in agreement with a temporal increase in TDR reaching moderate levels in the most important urban centers in the country. However, this result contrasts with another contemporary study including 85 individuals from Buenos Aires, Cordoba and Rosario enrolled in 2005–2007, which reported a low global TDR level of 2.4% [126]. Of note, a study in 35 ART-naïve pregnant women from Buenos Aires, performed between 2008 and 2011, reported 20.0% TDR to any ARV drug family [127]. Similarly, a recent study performing HIV genotyping in 12 pregnant women (among other types of populations) in 2008–2013 at a public hospital in Buenos Aires, reported high NNRTI TDR (16.7%) in this sub-group [128]. Additionally, a large study in Buenos Aires comparing two periods: 2003–2005 and 2006–2008 showed a clear increasing trend of TDR in time from 3.2% to 8.0% to any ARV drug [118].

**Fig 9:**
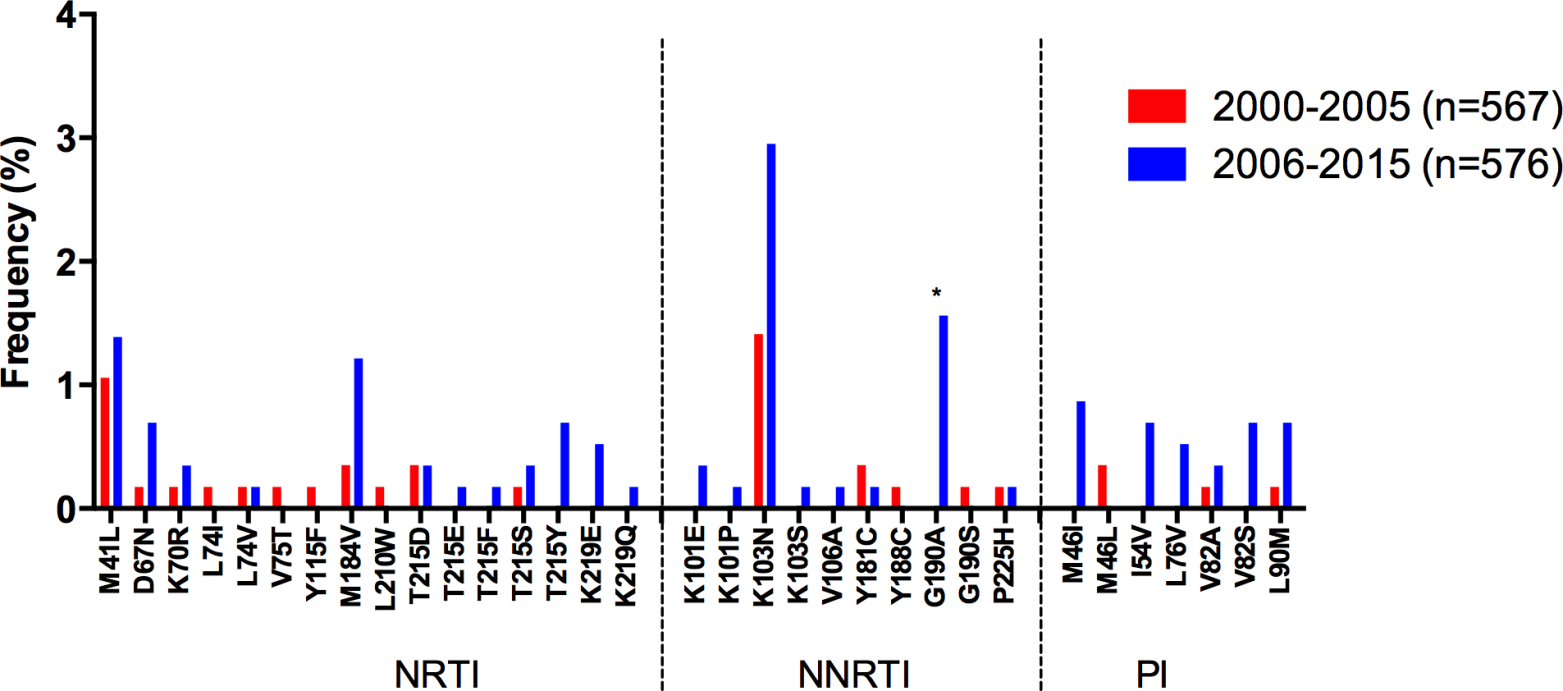
Drug resistance mutation frequency comparison, 2000–2005 vs. 2006–2015, for the Southern Cone. Drug resistance mutation frequency is shown for the studies included in the meta-analysis. Only surveillance mutations included in the WHO list are shown, ordered by drug family. *Significant differences (p<0.05), Fisher’s exact test. NRTI–nucleoside reverse transcriptase inhibitors; NNRTI–non-nucleoside reverse transcriptase inhibitors; PI–protease inhibitors.

The meta-analysis in the Southern Cone included 11 studies, encompassing 1,143 individuals, with an overall TDR estimation of 6.2 (95% CI: 4.9–7.8%; Table 2). A significant increase in TDR to any ARV drug was observed (p = 0.0068), which coincided with an increase in NNRTI TDR (p = 0.0008) and in particular of G190A mutation (p = 0.0038), while the increase in frequency for K103N was not significant (Fig 9). The most frequent DR mutations in the region were M41L (1.2%) and K103N (2.2%).

## Discussion

We have presented an updated review on HIV TDR in LAC, including 98 studies published between January 2000 and June 2015, encompassing 18,320 individuals. We have also performed a sub-regional TDR meta-analysis of all studies with available HIV sequences or individual DR mutation frequency data, including 81 publications and 11,441 individuals. LAC has currently the highest ART coverage among low and middle income countries; nevertheless, several factors have been recorded that could be associated with current TDR levels in the region [7]. Many of LAC universal access ART programs were introduced in the early ‘90s consisting of mono- and dual therapies. Also, it is estimated that by the end of 2013, approximately 24% of persons on ART were on second line regimens and 5% on third line regimens in LAC, representing 29% of people on ART having experienced treatment failure [7]. Also according to 2013 data, 23% of persons on ART in LAC had unsuppressed VL, with significant variation among countries ranging from 57% to 12% [7]. A wide range of drug stock-outs has been reported in LAC countries, ranging from 2 to 100% of establishments with stock-out episodes with an average duration of 17 days in 2012 [8]. The monitoring of HIVDR Early Warning Indicators provided important information on possible additional factors related to DR, including non optimized prescribing practices for ARV and adherence to national international guidelines, or individualized vs. public health optimized ARV prescribing approaches [9, 129].

In this meta-analysis, the frequency of TDR for the whole LAC remained at the moderate level (7.7%), in accordance to other recent reviews [12–15], reporting TDR in LAC from 1993 to 2013, with TDR prevalence ranging from 6.3% to 7.6%. In our study, TDR to each individual ARV drug class remained at the low level (<5%), as also reported by others [14, 15]. Also in agreement with previous studies [10, 14, 15], an increasing trend in global TDR, associated with NNRTI TDR was observed in the region when comparing the 2000–2005 and 2006–2015 periods (6.4% vs. 8.2%, p = 0.0007, for overall TDR and 1.9% vs. 4.2%, p<0.0001, for NNRTI TDR respectively). This observation is expected as the use of EFV-containing first line regimens has significantly increased in the region, with the most recent WHO survey reporting that nearly 69% of individuals were receiving first-line EFV-containing regimens including AZT+3TC+EFV (35%), TDF+3TC+EFV (18.2%), TDF+FTC+EFV (12.4%), and ABC+3TC+EFV (3.6%) in LAC [7]. Of note, nevirapine was still used in 13.6% of ART initiators. In agreement with data from WHO surveys, suggesting that greater ART coverage is associated with a higher TDR prevalence, particularly to NNRTI [10], Latin America shows the highest ART coverage rates as well as the highest TDR prevalence among regions, considering low-and middle income countries [15]. Temporal increases in NNRTI TDR were observed in most of the sub-regions, except for Mesoamerica. This increase was especially stark in the Caribbean and the Southern Cone, where NNRTI TDR reached moderate levels (8.1% and 5.6% respectively). Increasing NNRTI TDR was associated with increasing K103N frequency in most sub-regions, although evidence of increasing frequency of nevirapine-selected mutations [130] was also observed, particularly in the Caribbean, with significant increase of Y181C and G190A frequency. Indeed, a significant decrease in the frequency of these two mutations was observed in the Mesoamerica, possibly reflecting the current predominance of first line efavirenz-based regimens in this region.

Interestingly, a decreasing trend in NRTI TDR was also observed in LAC (4.7% vs. 3.8%, p = 0.02), possibly reflecting previous exposure to mono- or dual therapy and the consequent introduction of more potent ART schemes. The decrease in NRTI TDR was particularly evident in the Mesoamerican region (7.9% vs. 3.1%, p<0.0001), with a significant reduction in the frequency of M184V and of most thymidine analogue mutations (TAM), including D67N, K70R, L210W, T215F, T215Y, and K219E. This observation was mainly driven by two studies, both performed between 2002–2003: one in San Pedro Sula and Tegucigalpa, Honduras (n = 336), reporting NRTI TDR prevalence of 7.7% [34], and another in western Mexico (n = 100), reporting NRTI TDR prevalence of 13.0% [45], which caused an moderate NRTI TDR prevalence estimate in the 2000–2005 period. All Mesoamerican studies after 2005 included in the meta-analysis reported low levels of NRTI TDR.

We chose 2005 as the threshold year to compare TDR level between an older and a more recent study period. This year was important due to the UNAIDS “3 by 5” initiative, which aimed to provide ART to 3 million people in low- and middle-income countries by the end of 2005 [131]. Even when the global goal of 3 million people on ART was not achieved by 2005, most LAC countries had launched universal ART access programs, reaching 290,000 people on ART and a 65%-coverage according to ART guidelines applicable by that time [131, 132]. Thus, this year was an important milestone to mark the pre- and post-ART scale-up era in the region. Interestingly, all sub-regions reached moderate overall TDR levels in the more recent 2006–2015 period. However, overall TDR levels varied between low and moderate in the older 2000–2005 period, possibly consistent with different times of ART scale-up. An increase from low to moderate level was observed in the Caribbean, the Andean region and the Southern Cone.

Different programmatic factors could have different impacts on TDR at the sub-regional and/or the national level. Considering the national cascade of the continuum of HIV care, the percentage of total HIV-infected persons achieving viral suppression is low in some LAC countries such as Guatemala (19%), El Salvador (22%), Jamaica (12%), and Nicaragua (14%); while ARV drug stock-outs have been reported in many Caribbean countries (Bahamas, Barbados, Grenada, Saint Vincent and the Grenadines), and Panama [7]. Of note, in 2013, 13 countries were still prescribing one or more WHO non-recommended ARV drugs [7]. In particular, the rapid and marked increase in TDR to all ARV drug classes observed in the Caribbean is worrisome and underscores the need for strengthening health systems in hand with robust EWI monitoring and HIVDR surveillance.

Published data on estimated prevalence of HIV TDR have been generated either from routine information from clinical practice, prospective cohort studies, clinical trials, or TDR surveys. Comparing data across these sources has certain limitations, mainly due to methodological factors influencing the final prevalence estimate, such as study design and sampling method, sample size, type of population and survey sites (e. g. general population attending voluntary counseling and testing sites, pregnant women at antenatal clinics, blood donors, patients starting ART at HIV clinics, special populations such as men who have sex with men, female sex workers, people who inject drugs, inmates), selection criteria of study participants (e. g. recent vs. chronic infection), geographical context (e. g. urban vs. rural), as well as the method and classification criteria of TDR mutations. In the meta-analysis of the present work, 81 studies were combined including all types of populations, time of infection (chronic vs. recent), and study designs. Also, TDR analysis criteria were unified using the WHO list of mutations for TDR surveillance. In order to have adequate and comparable data on HIV TDR across countries and regions, as well as within the same country over time, the WHO has developed a standard list of TDR mutations to provide a common reference for correct and homogeneous classification of mutations [18], additionally from the Stanford HIVDR algorithm. With a total of 11,441 individuals included, population-specific effects could be masked by general population trends, making our retrospective observations relevant in a sub-regional level. Of note, several countries were under-represented or lacking, warranting the implementation of TDR surveys in these areas. In particular, surveys in the Caribbean only included Cuba, Dominican Republic and Jamaica, with most of the countries in the region lacking representation. In Latin America, Costa Rica, Ecuador, Bolivia, Guyana, Suriname, French Guiana, Uruguay, and Paraguay were notably lacking. Also of note was the lack of recent surveys in some areas or countries, which further limits our conclusions on TDR trends. This is the case of the Andean region and the Caribbean, with most studies being older than five years (S1 Table). Given the high conceptual and methodological heterogeneity of HIVDR studies, the implementation of HIVDR surveys based on a standardized methodology and with national representativeness, as recommended by the WHO, is warranted to generate reliable data that could be used to inform public health policies, especially in relation to ARV selection for first- and second-line and use of HIV genotyping for clinical monitoring. Moreover, these data would be comparable between different countries and overtime, giving a better perspective on the evolution of HIVDR regionally and globally.

## Conclusions

This review and meta-analysis provides an updated integration of data available on HIV TDR in LAC from 2000 to 2015. In accordance with other recent reviews, the overall levels of TDR remain at the moderate level, with a significant temporal increase in NNRTI TDR, consistent with the dominant use of EFV-containing first-line ART regimens in the region based on current WHO recommendations. Sub-regional scenarios varied, with all regions eventually reaching moderate TDR levels to any ARV drug class after the implementation of universal access programmes to ART. The rapid increase in TDR to all ARV drug classes in the Caribbean is notable, as well as the significant temporal increase in NNRTI TDR reaching moderate levels in the Caribbean and the Southern Cone.

Even acknowledging the limitations of this analysis, the observed increasing trend in NNRTI TDR supports the need to strengthen HIVDR surveillance based on standardized methodologies and programme monitoring and evaluation, in particular EWI and HIV care and treatment cascade analysis in all countries, to better characterize the continuum of HIV care and identify gaps and factors that may be corrected to prevent further emergence and transmission of resistance. Surveillance and prevention of drug resistance should be a critical component of any national programmatic response to HIV, to ensure the long-term effectiveness and sustainability of ART and the achievement of the 90-90-90 targets by 2020.

## Supporting Information

S1 Appendix Summary of studies included in the meta-analysis. (XLSX)

S1 Table PDR meta-analysis for the LAC region, 2000–2015, dividing into three sampling periods. (DOCX)

S2 Table PRISMA Checklist. (DOCX)

S1 Text Search terms. (DOCX)
