# Supplementary material for: Surveillance of HIV Transmitted Drug Resistance in Latin America and the Caribbean: A Systematic Review and Meta-Analysis
Source: PLoS One. 2016 Jun 29;11(6):e0158560. doi: 10.1371/journal.pone.0158560 (PMC4927069; doi:10.1371/journal.pone.0158560)
Supplement: S1 Table — (DOCX) [file pone.0158560.s002.docx]

Table S1. PDR meta-analysis for the LAC region, 2000-2015 dividing into three sampling periods

|  | Complete cohort | | 2000-2005 | | 2006-2010 | | 2011-2015 | | p value ^a^ |
| --- | --- | --- | --- | --- | --- | --- | --- | --- | --- |
|  | % | (95% CI) | % | (95% CI) | % | (95% CI) | % | (95% CI) |  |
| Caribbean | n=1,004 | | n=415 | | n=510 | | n=79 | |  |
| Any ARV Drug | 8.5 | (6.8, 10.4) | 3.6 | (2.0, 5.9) | 12.2 | (9,4, 15.3) | 10.1 | (4.5, 19.0) | 0.0192 |
| NRTI | 5.4 | (4.1, 7.0) | 3.4 | (1.9, 5.6) | 7.7 | (5.5, 10.3) | 1.3 | (0.0, 6.9) | NS |
| NNRTI | 4.9 | (3.6, 6.4) | 0.2 | (0.0, 1.3) | 8.0 | (5.8, 10.7) | 8.9 | (3.6, 17.4) | <0.0001 |
| PI | 0.8 | (0.3, 1.6) | 0.0 | (0.0, 0.9)) | 1.6 | (0.7, 3.1) | 0.0 | (0.0, 4.6) | NS |
| Mesoamerica | n=3,663 | | n=560 | | n=1958 | | n=1145 | |  |
| Any ARV Drug | 7.3 | (6.5, 8.2) | 9.3 | (7.0, 12.0) | 6.9 | (5.8, 8.1) | 7.2 | (5.7, 8.8) | NS |
| NRTI | 3.8 | (3.2, 4.5) | 7.9 | (5.8, 10.4) | 3.9 | (3.1, 4.9) | 1.7 | (1.1, 2.7) | <0.0001 |
| NNRTI | 3.5 | (3.0, 4.2) | 5.0 | (3.3, 7.1) | 2.4 | (1.8, 3.2) | 4.8 | (3.6, 6.2) | NS |
| PI | 1.4 | (1.1, 1.9) | 1.6 | (0.7, 3.0) | 1.6 | (1.1, 2.2) | 1.0 | (0.5, 1.8) | NS |
| Andean | n=667 | | n=410 | | n=267 | | n=0 | |  |
| Any ARV Drug | 5.7 | (4.1, 7.7) | 3.9 | (2.2, 6.3) | 8.2 | (5.2, 12.2) |  |  | 0.0251 |
| NRTI | 3.0 | (1.8, 4.6) | 2.4 | (1.2, 4.4) | 3.7 | (1.8, 6.8) |  |  | NS |
| NNRTI | 2.1 | (1.2, 3.5) | 1.0 | (0.3, 2.5) | 3.7 | (1.8, 6.8) |  |  | 0.0233 |
| PI | 2.2 | (1.3, 3.7) | 2.0 | (0.8, 3.8) | 2.6 | (1.1, 5.3) |  |  | NS |
| Brazil | n=4,954 | | n=1548 | | n=2834 | | n=572 | |  |
| Any ARV Drug | 8.4 | (7.7, 9.2) | 6.7 | (5.5, 8.1) | 9.2 | (8.1, 10.3) | 9.3 | (7.0, 11.9) | NS |
| NRTI | 4.4 | (3.8, 5.0) | 4.9 | (3.8, 6.0) | 4.4 | (3.7, 5.2) | 2.8 | (1.6, 4.5) | NS |
| NNRTI | 3.5 | (3.0, 4.0) | 1.2 | (0.7, 1.9) | 4.3 | (3.6, 5.1) | 5.4 | (3.7, 7.6) | <0.0001 |
| PI | 2.1 | (1.7, 2.6) | 1.8 | (1.2, 2.6) | 2.3 | (1.8, 3.0) | 1.9 | (1.0, 3.4) | NS |
| Southern Cone | n=1,143 | | n=567 | | n=288 | | n=288 | |  |
| Any ARV Drug | 6.2 | (4.9, 7.8) | 4.2 | (2.7, 6.2) | 6.9 | (4.3, 10.5) | 9.4 | (6.3, 13.3) | 0.0036 |
| NRTI | 2.7 | (1.9, 3.8) | 2.5 | (1.4, 4.1) | 3.5 | (1.7, 6.3) | 2.4 | (1.0, 4.9) | NS |
| NNRTI | 3.7 | (2.7, 4.9) | 1.8 | (0.8, 3.2) | 4.5 | (2.4, 7.6) | 6.6 | (4.0, 10.1) | 0.0005 |
| PI | 1.4 | (0.8, 2.3) | 0.7 | (0.2, 1.8) | 2.4 | (1.0, 4.9) | 1.7 | (0.6, 4.0) | NS |
| Complete Region | n=11,441 | | n=3500 | | n=5590 | | n=2351 | |  |
| Any ARV Drug | 7.7 | (7.2, 8.2) | 6.0 | (5.3, 6.9) | 8.5 | (7.8, 9.3) | 8.1 | (7.1, 9.3) | 0.0023 |
| NRTI | 4.0 | (3.7, 4.4) | 4.5 | (3.8, 5.2) | 4.5 | (4.0, 5.1) | 2.3 | (1.7, 2.9) | <0.0001 |
| NNRTI | 3.6 | (3.2, 3.9) | 1.8 | (1.4, 2.3) | 4.0 | (3.5, 4.5) | 5.2 | (4.3, 6.2) | <0.0001 |
| PI | 1.7 | (1.5, 2.0) | 1.4 | (1.0, 1.8) | 2.0 | (1.7, 2.4) | 1.5 | (1.0, 2.1) | NS |

^a^ Fisher’s exact or Chi-square test comparing the 2000-2005 vs. 2011-2015 periods, except for the Andean region, where 2000-2005 vs. 2006-2011 are compared. PDR, pre-antiretroviral treatment drug resistance; ARV, antiretroviral; NRTI, nucleoside reverse transcriptase inhibitors; NNRTI, non-nucleoside reverse transcriptase inhibitors; PI, protease inhibitors; CI, confidence interval; NS, non significant (p>0.05).
