# Supplementary material for: Surveillance of HIV Transmitted Drug Resistance in Latin America and the Caribbean: A Systematic Review and Meta-Analysis
Source: PLoS One. 2016 Jun 29;11(6):e0158560. doi: 10.1371/journal.pone.0158560 (PMC4927069; doi:10.1371/journal.pone.0158560)
Supplement: S1 Text — (DOCX) [file pone.0158560.s004.docx]

The following search terms were used:

(HIV OR AIDS OR “HUMAN IMMUNODEFICIENCY SYNDROME” OR “ACQUIRED IMMUNODEFICIENCY SYNDROME”) AND (resistance OR “drug resistance” OR genotypic) AND (“Latin America*” OR “South America” OR “Central America” OR Caribbean OR Anguilla OR “Antigua and Barbuda” OR Argentina OR Aruba OR Bahamas OR Barbados OR Belize OR Bermuda OR Bolivia* OR Brazil* OR “British Virgin Islands” OR “Cayman Islands” OR Chile* OR Colombia* OR “Costa Rica” OR Cuba* OR Dominica OR “Dominican Republic” OR “El Salvador” OR Ecuador OR “French Guiana” OR Grenada OR Guadalupe OR Guatemala OR Guyana OR Haiti OR Honduras OR Jamaica* OR Martinique OR Mexico OR Montserrat OR “Netherlands Antilles” OR Nicaragua* OR Panama OR Paraguay* OR Peru OR “Puerto Rico” OR “Saint Kitts and Nevis” OR “Saint Lucia” OR “Saint Vincent and the Grenadines” OR Suriname OR “Trinidad and Tobago” OR “Turks and Caicos Islands” OR Uruguay* OR Venezuela OR brasil* OR Argentinean OR Mexican OR costaric* OR “the Valley” OR “Saint John's” OR “Buenos Aires” OR Basseterre OR Basse-Terre OR Hamilton OR OR Oranjestad OR Nassau OR Bridgetown OR Belmopan OR Sucre OR “La Paz” OR Brasilia OR “Sao Paulo” OR “Rio de Janeiro” OR “Belo Horizonte” OR Maceió OR Manaus OR Goiânia OR Belem OR “Porto Alegre” OR Florianopolis OR “George Town” OR Santiago OR Valparaiso OR Concepción OR Temuco OR Bogota OR Cali OR Medellin OR Barranquilla OR “San Jose” OR Havana OR Habana OR “Santo Domingo” OR Roseau OR “San Salvador” OR Quito OR Guayaquil OR “Saint George's” OR “Port au Prince” OR “Port of Spain” OR Tegucigalpa OR Kingston OR Kingstown OR Willemstad OR Managua OR Asuncion OR Lima OR “San Juan” OR Marigot OR Castries OR Paramaribo OR Montevideo OR Caracas OR “Road Town” OR Mexicali OR “Tuxtla Gutiérrez” OR Chihuahua OR Guanajuato OR Guadalajara OR Toluca OR Morelia OR Monterrey OR Puebla).
